# RNA knowledge-graph analysis through homogeneous embedding methods

**DOI:** 10.1093/bioadv/vbaf109

**Published:** 2025-05-13

**Authors:** Francesco Torgano, Mauricio Soto Gomez, Matteo Zignani, Jessica Gliozzo, Emanuele Cavalleri, Marco Mesiti, Elena Casiraghi, Giorgio Valentini

**Affiliations:** AnacletoLab, Dipartimento di Informatica, Università degli Studi di Milano, Milan 20133, Italy; AnacletoLab, Dipartimento di Informatica, Università degli Studi di Milano, Milan 20133, Italy; CONNETS, Dipartimento di Informatica, Università degli Studi di Milano, Milan 20133, Italy; AnacletoLab, Dipartimento di Informatica, Università degli Studi di Milano, Milan 20133, Italy; AnacletoLab, Dipartimento di Informatica, Università degli Studi di Milano, Milan 20133, Italy; AnacletoLab, Dipartimento di Informatica, Università degli Studi di Milano, Milan 20133, Italy; AnacletoLab, Dipartimento di Informatica, Università degli Studi di Milano, Milan 20133, Italy; ELLIS—European Laboratory for Learning and Intelligent Systems, Università degli Studi di Milano, Milan 20133, Italy; BBOP Lab, Lawrence Berkeley National Laboratory, Berkeley, CA 94720, United States; KEPAKO Lab, Department of Computer Science, Aalto University, Espoo 02150, Finland; AnacletoLab, Dipartimento di Informatica, Università degli Studi di Milano, Milan 20133, Italy; ELLIS—European Laboratory for Learning and Intelligent Systems, Università degli Studi di Milano, Milan 20133, Italy

## Abstract

**Motivation:**

We recently introduced RNA-knowledge graph (KG), an ontology-based KG that integrates biological data on RNAs from over 60 public databases. RNA-KG captures functional relationships and interactions between RNA molecules and other biomolecules, chemicals, and biomedical concepts such as diseases and phenotypes, all represented within graph-structured bio-ontologies. We present the first comprehensive computational analysis of RNA-KG, evaluating the potential of graph representation learning and machine learning models to predict node types and edges within the graph.

**Results:**

We performed node classification experiments to predict up to 81 distinct node types, and performed both generic- and specific-edge prediction tasks. Generic-edge prediction focused on identifying the presence of an edge irrespective of its type, while specific-edge prediction targeted specific interactions between ncRNAs, e.g. between microRNAs (miRNA-miRNA) or between small interfering RNA-messenger and RNA-messenger molecules (siRNA-mRNA), or relationships between ncRNA and biomedical concepts, e.g. miRNA-disease or lncRNA-Gene Ontology term relationships. Using embedding methods for homogeneous graphs, such as Large-scale Information Network Embedding (LINE) and node2vec, in combination with machine learning models like decision trees and random forests, we achieved balanced accuracy exceeding 90% for the 20 most common node types and over 80% for most specific-edge prediction tasks. These results show that simple embedding methods for homogeneous graphs can successfully predict nodes and edges of the RNA-KG, paving the way to discover novel ncRNA interactions and laying the foundation for further exploration, and utilization of this rich information source to enhance prediction accuracy and support further research into the “RNA world.”

**Availability and implementation:**

Python code to reproduce the experiments is available at https://github.com/AnacletoLAB/RNA-KG_homogeneous_emb_analysis

## 1 Introduction

Knowledge graphs (KGs) have been widely applied in biomedicine to collect and integrate diverse data types with concepts grounded in biomedical ontologies (Nicholson and Greene 2020, Chandak *et al.* 2023).

We recently introduced RNA-KG, an ontology-based KG that integrates biological knowledge about coding and non-coding RNAs from more than 60 public databases (Cavalleri *et al.* 2024). RNA-KG incorporates functional relationships with genes, proteins, and chemicals, as well as ontologically grounded biomedical concepts. It was specifically designed to serve as input for artificial intelligence (AI)-based techniques aimed at inferring novel knowledge about RNA molecules, thereby supporting RNA-drug design.

The structure and information encoded in KGs can be leveraged by graph representation learning (GRL) techniques to infer new knowledge. GRL encompasses a class of machine learning methods that encode graph-structured data as vectors, referred to as embeddings, while preserving the graph’s structural, relational, and attribute-based properties (Xia *et al.*2021). These embeddings enable downstream tasks such as ‘link/edge prediction,’ which identifies novel associations between concepts (nodes) in the graph, and ‘node/edge-type prediction,’ which classifies node or edge types to reveal the structural organization of biological entities and their interactions. These techniques aid in identifying data patterns (Yi *et al.* 2022).

In this work, we present the first in-depth analysis of RNA-KG, employing homogeneous GRL techniques to determine whether relatively simple methods can effectively exploit the topological structure of local and global node neighborhoods to infer reliable knowledge from the KG and its biologically relevant subgraphs (views) extracted from RNA-KG.

More specifically, we first applied GRL methods to predict up to 81 distinct node types. Next, we performed ‘generic-edge prediction,’ which involves predicting the presence of any edge in the graph, irrespective of its type, where the edge type is determined by the types of the connected vertices. Finally, we employed GRL techniques for ‘specific-edge prediction,’ focusing on edges representing particular interactions between RNA molecules or relationships between RNA molecules and biomedical concepts.

The motivation behind conducting both edge prediction experiments lies in their distinct objectives and practical applications. The generic-edge prediction task evaluates whether the information encoded in the graph is sufficient and suitable for making accurate inferences, serving as a baseline assessment of the graph’s overall predictive power. However, in practical applications, the primary focus is often on determining whether specific nodes interact. For instance, one might seek to establish whether a particular small interfering RNA (siRNA) interacts with a target messenger RNA (mRNA) to induce RNA interference and knock down a specific gene (Damase *et al.* 2021). Similarly, specific miRNA-miRNA (microRNA) interactions are of interest, as an miRNA inhibitor could block the activity of another miRNA (Paunovska *et al.* 2022).

To address these practical needs, we introduced specific-edge prediction tasks, each performed on specific RNA-KG views. These tasks enable more targeted predictions, such as inferring relationships between an miRNA and a disease by leveraging the disease ontologies included in RNA-KG.

## 2 Methods

### 2.1 RNA-KG

RNA-KG (Cavalleri *et al.* 2024) is a KG that combines the publicly available information from more than 60 databases to obtain a centralized, uniform, and semantically consistent representation of the “RNA world.” These molecules are widely studied because they have a primary role in biological processes and pathways, especially those that are altered in cancer, genetic disorders, neurodegenerative diseases, cardiovascular conditions, and infections (Damase *et al.* 2021). The study of RNA is also one of the most promising avenues of research in therapeutics, as evidenced by the recent success of mRNA-based vaccines for the COVID-19 pandemic (Barbier et al. 2022). RNA-KG represents the existing knowledge about interactions involving RNA molecules and their interactions with other biomolecular data as well as with chemicals, diseases, abnormal phenotypes, and proteins to support the study and the discovery of the biological role of the “RNA world.”

Supplementary Table S1 provides detailed distributions of node types and edge types, highlighting the most prevalent categories in RNA-KG.

In this article, we use the undirected, unweighted version of RNA-KG.

Among the three classification tasks described in Section 3, the specific-edge prediction experiments were conducted on seven ‘RNA-KG views.’ Each view corresponds to a subgraph of the original RNA-KG, induced by selected node and edge types, and further enriched by integration with relevant portions of PheKnowLator’s Human Disease Benchmark KG (Callahan *et al.* 2024, PheKnowLator Ecosystem Developers 2024). This integration enabled the inclusion of additional biologically meaningful relationships, e.g., ‘gene-disease,’ ‘disease-disease,’ and ‘gene-gene’ associations, thereby enhancing the biomedical content of each view and making them suitable for specific prediction tasks of biological relevance.

More specifically, some views focus on the representation and inference of knowledge involving miRNA molecules, genes, aberrant phenotypes, and diseases (miRNADisease and miRNADiseaseSO views). Others, such as the GODisease view, are designed to support the exploration of underlying disease mechanisms. The piRNADisease and piRNADiseaseGO views aim to uncover the emerging role of piRNA molecules in diseases by relating them to molecular functions and biological processes. The ChemicalDisease view is particularly relevant for investigating how chemical exposures influence disease development, treatment, and progression. Finally, the cellAnatomy view emphasizes the spatial organization of biological entities, including proteins, genes, and RNA molecules—across cellular and anatomical structures. This enables the study of cell-specific gene expression, tissue-specific regulatory patterns, and the prediction of anatomical contexts for biomolecules. Further details of each view are reported in the Supplementary Information.

**Figure 1.**
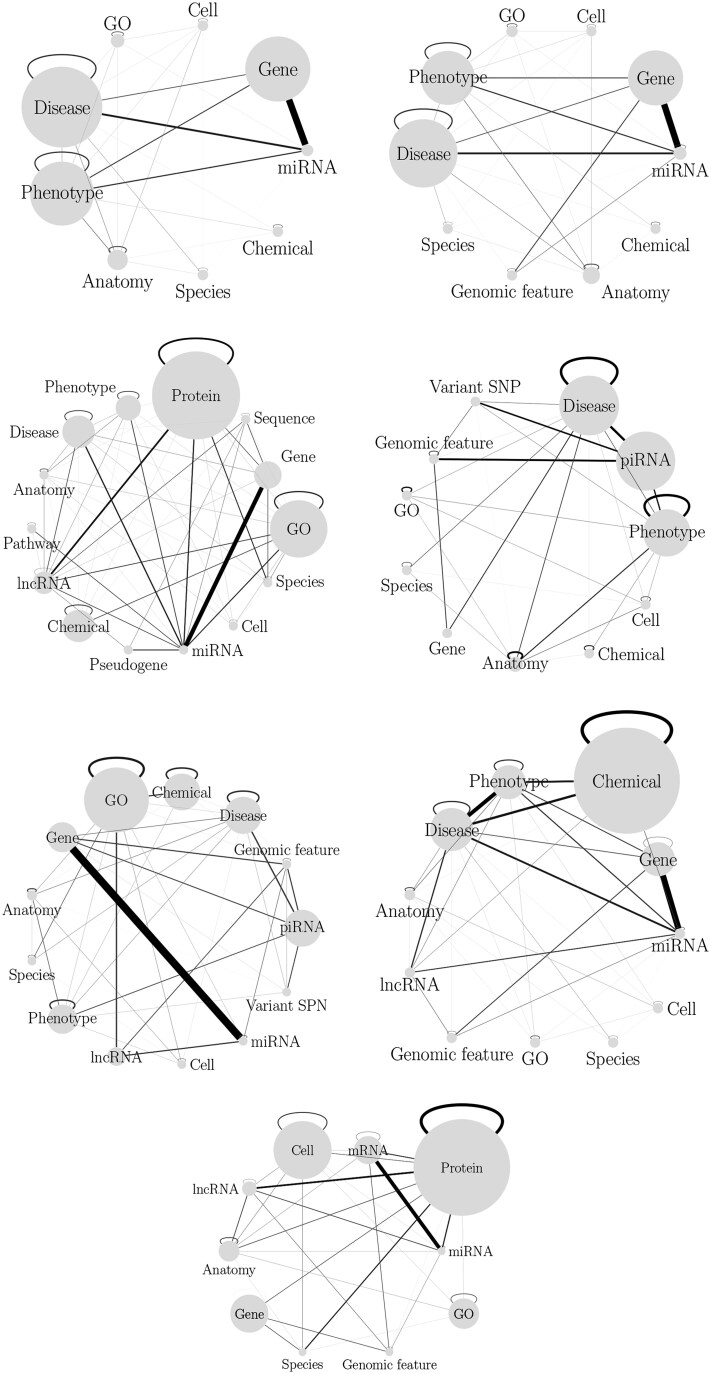
View schema. Each view is represented by a graph where node size is related to the number of nodes in the graph, while edge width and color represent the proportion of edges between nodes of the respective types. In the figure, we omitted node types accounting for less than 1% of the graph nodes.

Figure 1 depicts the schema of the views, and in Supplementary Section S3, we provide the rationale and motivation behind each view as well as detailed statistics describing their topology, including node-type and edge-type distributions, as well as Complementary Cumulative Distribution Function plots for distinct node types within each view. These statistics offer a comprehensive overview of the structure and content of the views, highlighting their utility in addressing biologically relevant classification tasks, such as miRNA-disease or piRNA-disease prediction.

### 2.2 Homogeneous GRL

Given a graph G=(V,E), where *V* is the set of nodes and *E* the set of edges, homogeneous GRL techniques learn a function $f:G\to R^{n}$ that maps (embeds) each node v∈V to a vector (alias, embedding) $x\in R^{n}$ such that nodes that are “close” in the graph *G* are also close in the vectorial space $R^{n}$, irrespective of their type. In other words, the embedding representation *x* of the node *v* preserves the topological characteristics of the node *v* in the embedding space $R^{n}$. These embeddings are then used by machine learning models to predict either the node/edge type or the existence of an edge.

Homogeneous GRL methods may be classified into the following three categories.

- Random walk-based methods for homogeneous graphs like DeepWalk (Perozzi *et al.* 2014), Walklets (Perozzi et al. 2016), or node2vec (Grover and Leskovec 2016) that use random walks on the graph to generate sequences of nodes that reflect the local and global connectivity patterns within the graph.
- Sampling-based methods that try to preserve the proximity between the nodes, like LINE (Tang *et al.* 2015) that uses edge sampling methods to improve performance during the optimization of the objective function, designed to preserve both local and global graph structures,
- Graph neural network-based methods like GraphSAGE (Hamilton *et al.* 2017) or Graph Attention Networks (Abu-El-Haija *et al.* 2018) can also be used to generate node embeddings. Graph neural networks showed excellent performance in graph prediction tasks, but require more complex and computationally intensive models (Ma *et al.* 2022).

Although homogeneous GRL methods simplify the embedding process by neglecting node and edge type information, they have proven their effectiveness and efficiency in inferring information from graph-structured data. In particular, by solely relying on the network topology while ignoring node/edge types, homogeneous GRL techniques are less computationally demanding than heterogeneous methods, which generally process individual types independently and then require an integration phase to pool the obtained representations (Dong *et al.* 2017). This makes homogeneous methods more suitable for large-scale graphs. Moreover, they avoid issues such as type imbalance that can arise in heterogeneous techniques, where the most represented types dominate the embeddings.

In this work, we investigate whether simple embedding methods can infer reliable knowledge from the RNA-KG or from smaller, biologically relevant, subgraphs (views) extracted from the overall graph. The two homogeneous embedding methods used in this study, node2vec (a random-walk-based method) and LINE (a sampling-based technique), were carefully selected based on their computational efficiency, simplicity, and proven performance. LINE was chosen for its speed, which allows for rapid experimentation and testing, while node2vec was selected for its demonstrated effectiveness in diverse graph-prediction tasks. The embeddings by these methods are independent of the subsequent classification task, which can be performed using the embeddings as input to any supervised machine learning model, even as relatively simple as those we tested in our study: Decision Trees (DTs, Breiman *et al.* 1984), Random Forests (RFs, Breiman 2001). (Note that we also tested radial basis function support vector machines, which achieved results similar to RFs. Due to the superior robustness of RFs w.r.t. their hyperparameter settings and their fastest convergence, we preferred reporting RF results. All the models were implemented using Python—*scikit-learn* (Pedregosa *et al.* 2011) library.)

## 3 Results

In this section, we describe the results obtained by embedding the graph elements with LINE and node2vec (under the settings defined in Subsection 3.1), and then training DTs, and RFs on such embeddings for three classification tasks:

- Node-type prediction tasks (Subsection 3.2): this task is applied to the entire RNA-KG. First, the graph nodes are embedded by node2vec. Next, the resulting embeddings are partitioned into five stratified train and test holdouts. Each training set, composed of the embeddings for the training nodes, is used to train DT or RF classifiers for the node classification task, while the embeddings corresponding to the test nodes are used to evaluate the performance of the trained classifiers.
- Generic-edge prediction tasks (Subsection 3.4): this task is applied to the entire RNA-KG. First, test edges are removed from the graph by guaranteeing that the number of connected components in the training graph is unchanged (see Subsection 3.3); this results in training and test graphs that consist solely of existing (i.e. positive) edges. Negative edges are then sampled using node-degree aware sampling for both training and test sets, ensuring that the degree distributions of positive and negative edges remain comparable. After embedding the nodes in the training graphs, edge embeddings are generated for both positive (existent) and negative (nonexistent) edges by concatenating the embeddings of the nodes at each edge’s vertices. These edge embeddings are then used to train DTs and RFs on the training set, with performance evaluated on the test set.
- Specific-edge prediction tasks (Subsection 3.5): this task is applied individually to the RNA-KG views described in Section 2.1 and detailed in Supplementary Table S1. The training and test graphs are constructed in a way that is similar to the one used in the generic-edge prediction task, ensuring that the graph structure remains consistent and that connectivity is preserved. However, in this case, only edges of a specific type are used for training and evaluation of DTs and RFs. Importantly, in this task, particular care should be devoted to negative edges sampling to avoid information flow between the training and the test edge sets (see Subsection 3.3).

In all our experiments, we used balanced accuracy as the performance evaluation measure, i.e. the average of recall across all classes (see Supplementary Section S2). This metric was chosen because it provides a fair evaluation across all classes regardless of their imbalanced frequency, as is the case in the node-type prediction task. When dealing with the specific-edge prediction experiments, we also provided the F1 and weighted F1 scores (Supplementary Section S6).

### 3.1 Graph embedding methods

To embed the graph with LINE, we utilized both the first-order and second-order proximity versions provided by the GRAPE library (Cappelletti *et al.* 2023), using the default hyperparameters.

For node2vec, we adopted the skip-gram model as the shallow neural network architecture, with GRAPE default hyperparameters.

To ensure a fair comparison, we generated random walk samples across multiple experiments using the same setting, defined by the same values for the return_weight ($\frac{1}{p}$) and explore_weight ($\frac{1}{q}$) hyperparameters. More precisely, we compared values that emulate a depth-first visit (DFS-setting with $\frac{1}{p}=0.2$, $\frac{1}{q}=5$), a breadth-first visit (BFS-setting with $\frac{1}{p}=5$, $\frac{1}{q}=0.2$), or an unbiased first-order random walk according to a DeepWalk-like strategy (Perozzi *et al.* 2014) (Balanced-setting with $\frac{1}{p}=1$, $\frac{1}{q}=1$).

**Figure 2.**
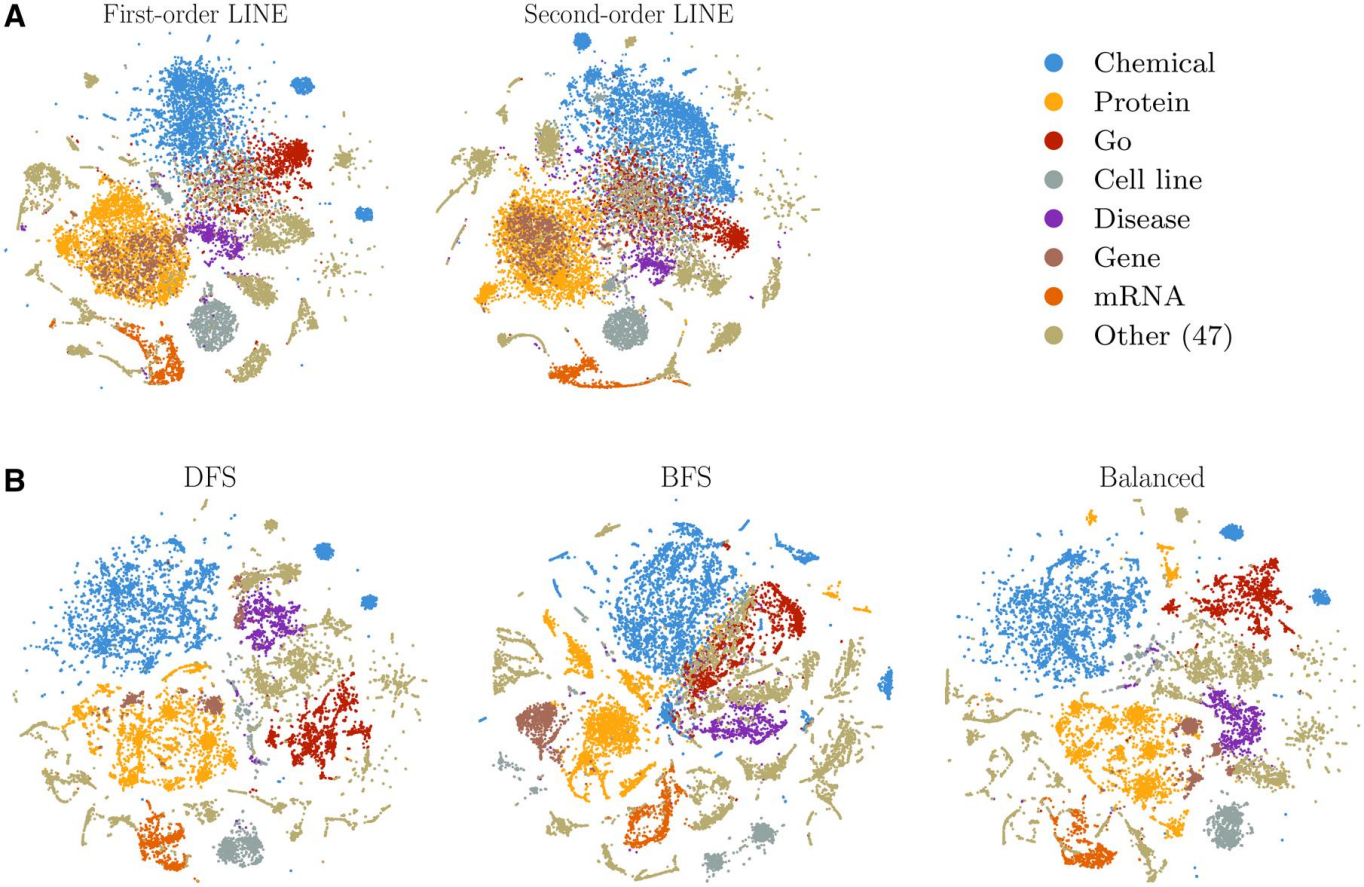
Two-dimensional t-SNE projections of node embeddings of the RNA-KG. (A) First (left) and second-order (right) LINE node embeddings; (B) node2vec with DFS-like (left) and BFS-like (center) hyperparameters and DeepWalk-like (right) node embeddings.

Figure 2 shows the t-SNE two-dimensional projections (van der Maaten and Hinton 2008) of the LINE and node2vec embeddings for the most frequent types of nodes in RNA-KG (see also the 2D tSNE embedding of less represented RNA types in Supplementary Fig. S11). The main categories of biomolecules and medical concepts are quite well separated, with some expected superpositions, such as the intersection between gene and protein embedded representations, because genes encode the information necessary to synthesize proteins. Our preliminary results showed that the BFS-like node2vec strategy performs slightly better in the predictive tasks compared to DFS-like (Supplementary Section S6 and Supplementary Fig. S13). This strategy has been used in all the experiments reported in Section 3.

### 3.2 Node-type prediction: experimental settings and results

For node-type prediction, we computed embeddings by using BFS-like node2vec to project points into multiple embedding sizes: 10, 50, and 100, and we also experimented with two-dimensional projections of 100-dimensional embeddings through t-SNE. On average, 10D embeddings achieved the best balance between predictive accuracy and efficiency (more details in Supplementary Section S4). The embeddings were input to DTs and RFs. To perform an unbiased evaluation, we applied five stratified holdout cross-validation (train:test ratio = 70:30, with fixed seed for the random number generator to guarantee comparable results across models) on the RNA-KG nodes (about 578K nodes). Hyperparameter selection for the classifier models was performed on the training set using a grid search strategy and internal cross-validation.

**Figure 3.**
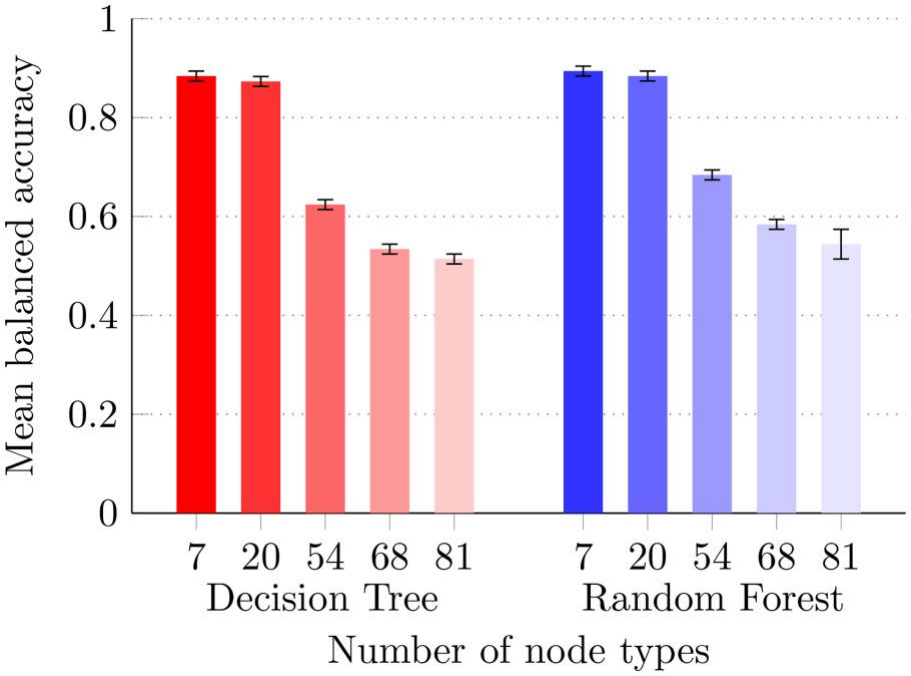
Balanced accuracy results on RNA-KG node type prediction using full 10-D BFS-like node2vec embeddings with DTs and RFs, considering the 7, 20, 54, 68, and 81 most common node types. The color intensity of the bars reflects their balanced accuracy value and evidence a drop in performance as the number of node types increases (discussed in Section 4). Error bars represent the standard deviation across multiple holdouts.

Figure 3 shows the average balanced accuracy results obtained from the 10D embeddings. Overall results are surprisingly positive. Using RFs, we achieve a balanced accuracy of 97.4% when classifying the seven most represented node types. Even when considering the 20 main node types, the results remain well above 90%. When considering 81 different node types, the balanced accuracy drops to 53.7%; however, it is important to note that a random guess predictor would achieve a balanced accuracy of only about 1.2%. Details about the considered node types are available in Supplementary Section S1.

### 3.3 Edge prediction: unbiased sampling of train and test sets

**Figure 4.**
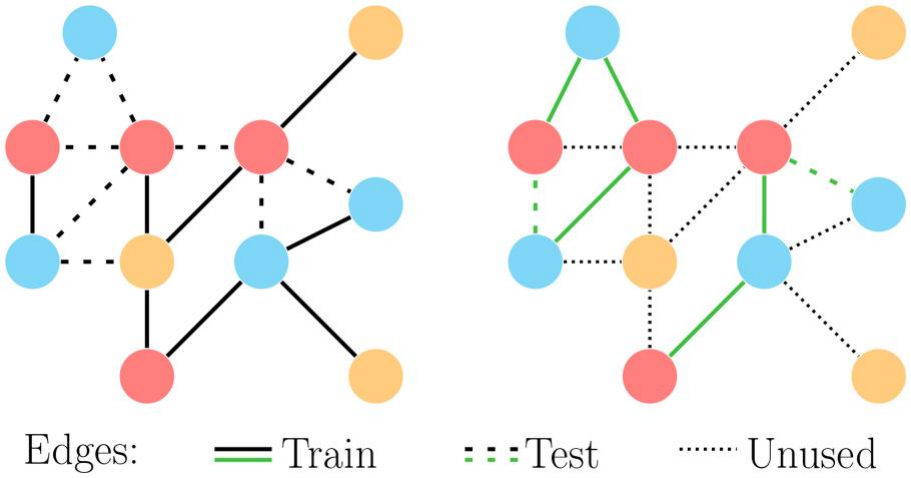
Generic- (left) and specific (right)-edge prediction, where the specific edges of interest are the green ones connecting blue and pink nodes. Continuous lines represent positive training edges, dashed ones represent positive test edges; dotted lines in the right panel represent the edges not used for either training or testing in the specific-edge prediction.

We experimented with generic- and specific-edge prediction tasks. Figure 4 depicts the main differences between these two types of edge prediction.

When performing edge prediction tasks, a commonly overlooked issue is the method used to create training and test edge samples.

The first important observation is that, since test edges must be removed from the input graph prior to graph embedding, their removal should not alter the graph’s global connectivity structure; specifically, we should not change the number of connected components. To ensure this, for both generic- and specific-edge prediction tasks, we generated training and test splits using a ‘Connected Monte-Carlo’ holdout scheme, which ensures that removing test edges does not increase the number of disconnected components compared to the original full RNA-KG (Cappelletti *et al.* 2023).

In the context of specific-edge prediction, particular attention should be given to the generation of negative edges, to avoid false negatives that can compromise the learning capabilities of the models. Indeed, if the graph used to generate the negative training edges is limited to the positive training graph, rather than the full graph, positive examples that are not included in the training set but that have been included in the test set could lead to false negatives that can confuse the predictor being trained, degrading its performance. This procedure, included in GRAPE, can be acceptable when we consider generic-edge prediction of the overall graph since the expected number of false negatives is relatively low, but with specific edges, the number of potential false negatives can be large, as we will show in the following experiments.

For this reason, we designed and implemented the following unbiased pipeline for specific-edge prediction:

- Generate the positive train $G_{tr}^{+}$ and positive test $G_{te}^{+}$ graph from the full graph *G*, while guaranteeing the connectivity and the same number of connected components in the positive train graph $G_{tr}^{+}$ as in the original graph *G*. This can be accomplished by using a spanning tree of *G* as $G_{tr}^{+}$, and $G_{te}^{+}$=G\$G_{tr}^{+}$.
- Generate the embeddings for the nodes of the graph by applying an embedding method on the training graph $G_{tr}^{+}$ (the embeddings are generated based on the train graph instead of the full graph to avoid bias in the results).
- Filter the positive train $G_{tr}^{+}$ and positive test graph $G_{te}^{+}$ edges by keeping only the specific edge type to obtain a filtered positive train $G_{tr}^{f+}$ and test graph $G_{te}^{f+}$.
- Generate the filtered negative graph $G^{f-}$ from the full graph *G* using node-degree aware edge sampling to guarantee that the sampled negative edges have degree-distribution comparable to the one characterizing positive edges (Cappelletti *et al.* 2024). In GRAPE, the negative test graph is generated from the filtered train graph $G_{tr}^{f+}$ and this can lead to a negative test graph that contains false negatives. This alternative method solves the problem.
- Split the filtered negative graph $G^{f-}$ into negative train graph $G_{tr}^{f-}$ and negative test graph $G_{te}^{f-}$ to avoid any intersection between the two sets.
- Train the edge prediction model using the filtered positive training graph $G_{tr}^{f+}$ and the filtered negative training graph $G_{tr}^{f-}$.
- Test the trained model with the filtered positive test graph $G_{te}^{f+}$ and the filtered negative test graph $G_{te}^{f-}$.

Details about the implementation of the unbiased train/test sampling approach can be found in Supplementary Section S7.

### 3.4 Generic-edge prediction

Generic-edge prediction experiments have been performed using five stratified holdout cross-validation, with a 70:30 train:test ratio, and using the same random seed generator to guarantee comparable results across models. Overall, the training set contains about 4M edges, and the test set with about 1.7M edges.

Both DTs and RFs hyperparameters are optimized by using grid-search on the training set (learning hyperparameters listed in Supplementary Section S5).

**Figure 5.**
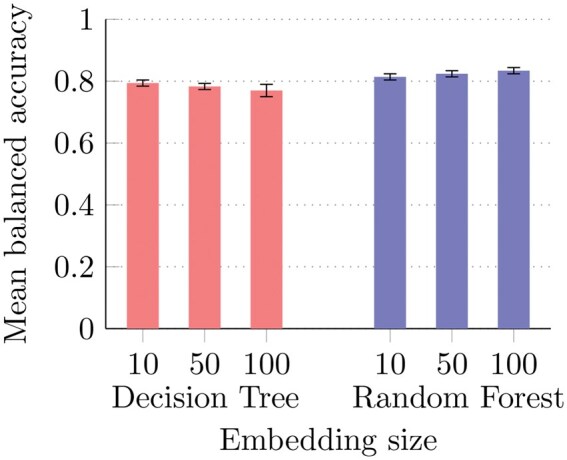
Balanced accuracy results for DT and RF on the RNA-KG dataset for the generic-edge prediction task, utilizing full BFS-like node2vec embeddings of various sizes. Vertical segments on each bar represent the standard deviation across multiple holdouts; note that in some cases the standard deviation is very low (<0.1%) and the segment is not visible.

Experimental results with different embedding dimensions are shown in Fig. 5. The embedding size has a certain impact on the Balanced Accuracy only with RFs. The best result is achieved with RFs trained on 100D embeddings. Again, results are encouraging and confirm the predictive capability of homogeneous embedding methods on RNA-KG.

### 3.5 Specific-edge prediction

For the specific-edge prediction tasks, we embedded each RNA-KG view by three embedding approaches, i.e. BFS and DFS-like node2vec Skip-Gram and first-order LINE, and trained RFs and DTs to classify the computed embeddings (all the experiments employed the same hyperparameter values, see Supplementary Section S6).

Generalization capabilities of the models were assessed using 5 random holdout cross-validation (train:test ratio = 70:30, with random seed generator set to a fixed value) in all the following experiments.

#### 3.5.1 Specific-edge prediction results with biased/unbiased train/test sampling

We compared the biased and unbiased train/test sampling approaches (Section 3.3) on a subset of RNA-KG views. The embeddings were generated using first-order LINE with embedding size set to 10 and the DT as classifier model.

Table 1 shows that the biased sampling can generate a consistent percentage of false negative edges (FN% column) in some specific-edge prediction tasks (e.g. 21% and 23% in, respectively, the miRNA-disease and miRNA-phenotype tasks). The performance of the classifier models is therefore consequently affected, as witnessed by the accuracy on the positive edges that drops to 50%. This issue is solved when using the model trained with the unbiased training pipeline; indeed, the accuracy on the positive edges improves to more than 75% in both cases. On the other hand, results on negative edges are not affected, thus resulting in boosted balanced accuracy. As expected, when the percentage of false negatives is low, the performance obtained when using the biased and unbiased sampling approaches are comparable (Table 1).

**Table 1.** Comparison of the biased and unbiased pipeline on the miRNA-disease view of the RNA-KG across six edge specific-prediction tasks.^a^

Specific-edge prediction	Unbiased procedure	FN (%)	Accuracy on pos.	Accuracy on neg.	Overall BA (%)
miRNA-disease	No	21.27	50.10 ± 0.21	75.31 ± 0.18	62.71 ± 0.11
miRNA-disease	Yes	0.00	75.98 ± 0.44	74.34 ± 0.42	75.16 ± 0.24
Disease-disease	No	0.17	48.34 ± 0.53	63.48 ± 0.29	55.91 ± 0.23
Disease-disease	Yes	0.00	47.34 ± 0.73	64.32 ± 0.57	55.83 ± 0.47
miRNA-gene	No	4.00	56.03 ± 0.12	59.14 ± 0.11	57.59 ± 0.08
miRNA-gene	Yes	0.00	59.36 ± 0.14	57.81 ± 0.11	58.59 ± 0.11
Gene-disease	No	0.51	52.39 ± 0.83	62.63 ± 0.75	57.51 ± 0.34
Gene-disease	Yes	0.00	51.30 ± 0.48	62.85 ± 1.38	57.07 ± 0.82
Gene-phenotype	No	1.26	53.55 ± 0.59	54.57 ± 0.55	54.06 ± 0.35
Gene-phenotype	Yes	0.00	53.34 ± 0.53	53.59 ± 0.52	53.46 ± 0.31
miRNA-phenotype	No	23.15	50.25 ± 0.36	77.20 ± 0.32	63.73 ± 0.17
miRNA-phenotype	Yes	0.00	77.01 ± 0.40	75.36 ± 0.18	76.19 ± 0.16

a FN stands for False negatives and BA for Balanced Accuracy.

#### 3.5.2 Specific-edge prediction results with the unbiased pipeline on different views of the RNA-KG

Finally, we conducted a thorough analysis of various views of the RNA-KG, each extracted from the overall RNA-KG to focus on specific types of nodes and edges relevant to a given edge prediction task.

**Figure 6.**
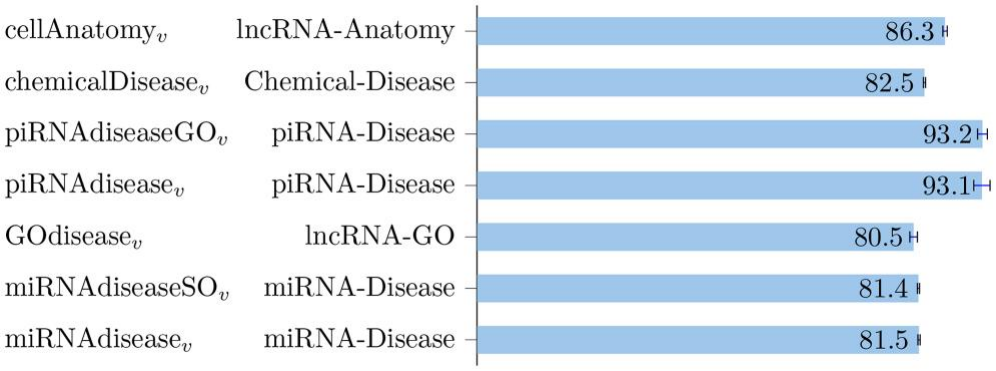
Mean balanced accuracy (and its standard deviation) across five holdouts, achieved by RFs on specific-edge prediction tasks performed over the RNA-KG views. The *y*-label reports the name of the view and the specific-edge prediction task.

The balanced accuracy results for the main specific-edge prediction tasks are summarized in Fig. 6. These results were obtained using a 10D LINE embedding combined with DTs and RFs.

Detailed results for specific-edge prediction tasks across seven RNA-KG views, along with performance metrics on a more challenging test set containing 10× times as many negative edges as positive edges, are provided in Supplementary Section S6.

## 4 Discussion

In this work, we conducted a systematic analysis of RNA-KG, an ontology-based KG describing RNA molecules, to assess its informativeness via thorough and unbiased prediction experiments using relatively simple homogeneous graph embedding techniques, such as node2vec and LINE. We obtained surprisingly good results, confirming that even graph embedding methods for homogeneous graphs can reasonably predict node types and edges of the RNA-KG. These results open the way to the application of more complex graph embedding methods aware of the heterogeneity of the underlying RNA-KG (M. Soto-Gomez et al, unpublished results for publication), and to end-to-end classification methods based on GNNs to further improve the prediction performance (Li et al. 2022).

Indeed, as shown in Fig. 3, the node-type prediction results yield high-balanced accuracy using relatively simple classifiers (DTs or RFs). While RFs tend to achieve better results than DTs, both the classifiers suffer from a drop in performance as the number of considered node types increases. This decrease is attributable to two factors: as the number of classes increases, the less represented classes tend to overlap with the larger ones, and the classification problem becomes significantly unbalanced, presenting an additional challenge for any classifier. This is further supported by the observation that when only the 7 or 20 most represented classes are considered, the standard deviation across the five holdouts is very low (less than 0.1%), highlighting that fewer classes result in less challenging classification tasks and more stable classifier performance. Of course, these results may be improved by using more complex, eventually heterogeneous, graph embedding strategies coupled with imbalance-aware classifiers (Schubach *et al.* 2017).

Full embeddings lead to significantly better results (Wilcoxon rank-sum test, *P* value $<10^{-5}$), which is expected since 2D projections overly compress the data and result in a loss of important features.

Additionally, for the generic-edge prediction task, RFs trained on embedded edges achieve accuracy greater than 80%, confirming that embedding methods for homogeneous graphs can reasonably predict the existence of an edge in RNA-KG.

Edge prediction tasks focusing on specific edges in RNA-KG, using views tailored to the prediction task, show that we can obtain accuracy larger than 80%. In several cases, such as piRNA-disease prediction, accuracy exceeds 90% (Fig. 6). This performance is achieved using relatively simple and fast embedding methods, such as LINE, in combination with off-the-shelf classifiers like DTs and RFs.

Overall, these results indicate that we can reasonably predict nodes and edges in RNA-KG using relatively simple embedding methods for homogeneous graphs, thereby demonstrating both the effectiveness of these methods and the high quality of the data available in RNA-KG. This is of paramount importance for discovering novel interactions between different types of noncoding RNA or other biomolecular entities, or for uncovering relationships between RNAs and specific biomedical concepts. Indeed, the rich representation of different RNA types in RNA-KG enables the discovery of novel interactions between RNA molecules, e.g. miRNA-mRNA, miRNA-miRNA, miRNA-lncRNA, and siRNA-mRNA, as well as interactions between RNA molecules and other biomolecules, such as aptamer-protein, lncRNA-protein, and siRNA-gene. Moreover, RNA-KG allows us to represent and uncover associations involving genes and diseases (gene-disease), molecular functions and processes (gene-GO), and various RNA-medical concept associations, including miRNA-disease, lncRNA-GO, and miRNA-pathway. Such new knowledge can be inferred by analyzing the embedded representations of negative edges, i.e. edges not present in RNA-KG, prioritizing those scoring high predicted probability. These edges connect nodes for which no known association currently exists; high-scoring predictions may indicate the presence of yet unknown interactions. Importantly, the discovery of novel RNA interactions and associations can support the development of RNA-based therapeutics (Sparmann and Vogel 2023). For example, identifying a novel silencing interaction between a miRNA and a cancer-associated mRNA could inform the design of an RNA drug that targets and silences that gene.

Our findings represent the first systematic analysis of RNA-KG using relatively simple embedding methods for homogeneous graphs.

We estimate that the success of these methods partially relies on the topological differences between node types. Indeed, as shown in Supplementary Table S1 in the Supplementary Information, all views exhibit a substantial variability in the mean degree across node types. This property could facilitate a characterization of node and edge types based on their neighborhoods, which can be exploited by homogeneous methods. These differences are even more pronounced when considering the semantic induced of node types. Therefore, we anticipate substantial improvements in prediction performance by employing methods that account for the rich semantic heterogeneity of the graph (Bing *et al.* 2023), thereby exploiting the available knowledge about the diverse types of nodes and edges in RNA-KG (Soto-Gomez *et al.* 2024). Furthermore, considering that RNA-KG is constructed by integrating ontologies that hierarchically organize information, we plan to explore hyperbolic embedding techniques, as hyperbolic spaces are known to better model hierarchical information compared to Euclidean spaces (Nickel and Kiela 2017, Sala *et al.* 2018).

## 5 Conclusion

In summary, our results show that GRL methods can accurately predict node types and edges in RNA-KG. The resulting list of predicted interactions and relationships could serve as an invaluable resource for guiding the experimental efforts of biomedical researchers, thus paving the way for novel discoveries and insights into the “RNA-world.”

## Supplementary Material

vbaf109_Supplementary_Data

## Data Availability

RNA-KG is available at https://rna-kg.anacleto.di.unimi.it/ and RNA-KG views are available at https://rna-kg.anacleto.di.unimi.it/views/. The RNA-KG version used is 0.2 which is also available to download from https://zenodo.org/records/10418431. The code that was used to run the experiments is available on GitHub (https://github.com/AnacletoLAB/RNA-KG_homogeneous_emb_analysis).
