# Supplementary material for: RNA knowledge-graph analysis through homogeneous embedding methods
Source: Bioinform Adv. 2025 May 13;5(1):vbaf109. doi: 10.1093/bioadv/vbaf109 (PMC12150776; doi:10.1093/bioadv/vbaf109)
Supplement: vbaf109_Supplementary_Data [file vbaf109_supplementary_data.pdf]

Supplementary Information

RNA Knowledge Graph analysis through homogeneous embedding methods

Contents

S1 RNA-KG node types **2**

S2 Balanced accuracy **4**

S3 RNA-KG views **4**
S3.1 miRNAdisease 6
S3.2 miRNAdiseaseSO 7
S3.3 GOdisease 8
S3.4 piRNAdisease 9
S3.5 piRNAdiseaseGO 11
S3.6 chemicalDisease 14
S3.7 cellAnatomy 16

S4 Node Type Prediction **17**

S5 Generic Edge Prediction **23**

S6 Specific Edge Prediction **25**
S6.1 MiRNAdisease View 28
S6.2 MiRNAdiseaseSO View 29
S6.3 GOdisease View 29
S6.4 PiRNAdisease View 32
S6.5 PiRNAdiseaseGO View 32
S6.6 ChemicalDisease View 32
S6.7 CellAnatomy View 36

S7 Implementation of the Unbiased Training Procedure **38**
S7.1 Updating the Training Function 39

S1 RNA-KG node types

Supplementary Table S1 lists all the node types available in RNA-KG along with their respective counts.

Table S1: Node distribution respect to the node type for RNA-KG.

Node	Count	Proportion	Mean degree
Chemical	150080	0.2595	4.1148
Protein	96197	0.1663	11.1001
GO	43869	0.0758	11.2065
Cell line	41807	0.0723	7.0619
Disease	23262	0.0402	7.9286
tRF	20897	0.0361	13.2522
mRNA	20624	0.0357	121.1773
Gene	20081	0.0347	4.9014
Phenotype	16865	0.0292	6.3985
Variant (SNP)	16091	0.0278	3.0458
Riboswitch	15805	0.0273	8.7789
lncRNA	15511	0.0268	92.1723
miRNA	14295	0.0247	145.3281
Anatomy	14203	0.0246	7.8024
tsRNA	10243	0.0177	15.3638
Pseudogene	9837	0.0170	27.1647
circRNA	9216	0.0159	48.0559
Small protein	8178	0.0141	3.0584
Vaccine	6246	0.0108	4.9922
Viral RNA	5654	0.0098	2.0428
ASO	2633	0.0046	2.0171
Pathway	2600	0.0045	2.5454
Cell	2368	0.0041	18.2247
Sequence	2363	0.0041	78.6022
Species	2148	0.0037	60.1378
snRNA	1803	0.0031	16.7598
tRNA	894	0.0015	258.6521
Glycan	797	0.0014	11.7465
snoRNA	583	0.0010	31.2727
otherRNA	565	0.0010	22.8053
Environment	398	0.0007	4.6533
rRNA	350	0.0006	5.4857
Chromosome	249	0.0004	4.1606
Environment exposure	171	0.0003	4.7310
Neuro behaviour	165	0.0003	2.6667
Food	163	0.0003	3.1288
Experimenta factor	145	0.0003	73.4138
Aptamer	138	0.0002	2.1594
s(i/h)RNA	117	0.0002	2.0342
ncRNA	115	0.0002	10.8522
Protein modification	91	0.0002	117.9011
gRNA	77	0.0001	2.0000
Mouse pathology	75	0.0001	5.6000
Medical action	72	0.0001	1.9861
Plant	48	0.0001	3.8542
viral_mRNA	39	0.0001	7.4359
NCI thesaurus	28	0.0000	3.0714
scaRNA	23	0.0000	24.4783
lincRNA	22	0.0000	3.5455
Histone modification	20	0.0000	3675.3000
Epigenetic modification	18	0.0000	13.7778
Human developmental stage	16	0.0000	4.9375
Mental functioning	14	0.0000	2.8571
RNA drug	13	0.0000	4.4615
mtRNA	10	0.0000	4.8000
retained_intron	8	0.0000	5.1250
unknown RNA	8	0.0000	14.6250
undefined	6	0.0000	17.5000
General medical science	6	0.0000	2.3333
CP	6	0.0000	2.0000
Mental disease	6	0.0000	2.0000
PCO	6	0.0000	3.1667
Ribozyme	6	0.0000	983.6667
Y_RNA	4	0.0000	3.0000
Adverse events	4	0.0000	1.7500
eRNA	3	0.0000	2.6667
vRNA	3	0.0000	5.0000
ribozyme	3	0.0000	36.6667
ExO	3	0.0000	61.3333
scRNA	3	0.0000	36.0000
Biological role	3	0.0000	57.0000
snomedct	2	0.0000	10938.5000
Infectious disease	2	0.0000	1.0000
misc_RNA	2	0.0000	2.0000
TEC	2	0.0000	2.0000
Basic formal	1	0.0000	1113.0000
Trait	1	0.0000	2.0000
UMLS	1	0.0000	2.0000
Drosophila anatomy	1	0.0000	2.0000
piRNA	1	0.0000	2.0000
owlNothing	1	0.0000	208.0000

Figure S1: RNA-KG schema. In the graph, node size represent the proportion of nodes in the graph while edge width and color represent the proportion of edges between nodes of the respective types. In the figure we omitted node types accounting for less than 0.15% of the graph nodes.

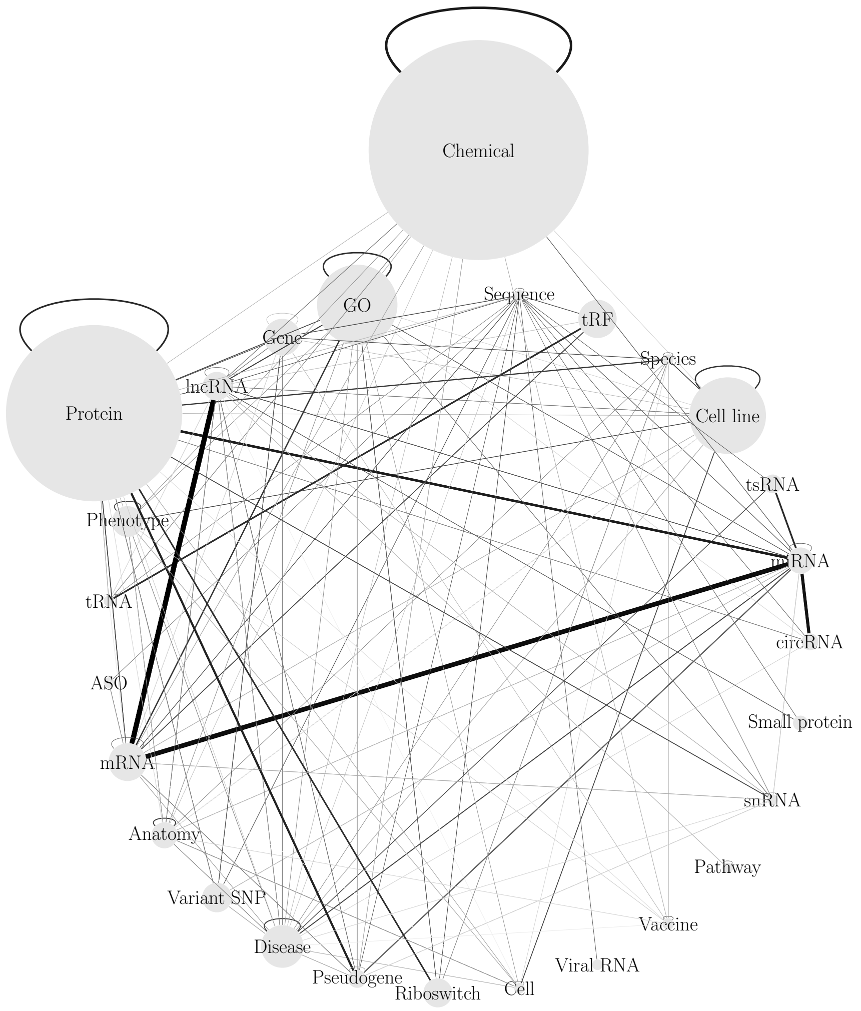

S2 Balanced accuracy

Balanced accuracy is a metric commonly used to evaluate binary or multi-class classification models, particularly when dealing with imbalanced datasets. It is defined as the average of the recall (also known as sensitivity or true positive rate) across all classes. Mathematically, for a multi-class classification problem with C classes, balanced accuracy is computed as:

$$\text{Balanced Accuracy} = \frac{1}{C}\sum_{i=1}^{C}\frac{\text{TP}_i}{\text{TP}_i + \text{FN}_i}$$

where:

- TP_i (true positives) represents the number of correctly classified instances of class i,
- FN_i (false negatives) represents the number of misclassified instances that belong to class i.

Note that in our experiments, and particularly for the node-type prediction task, we report Balanced Accuracy and avoid Weighted F1-scores to provide a fair evaluation of performance across all classes, regardless of their frequency. Weighted F1 is heavily influenced by the most represented classes, which can lead to overly optimistic results in imbalanced settings, as it averages class-wise F1-scores using class prevalence as weights. In contrast, Balanced Accuracy computes the average recall per class, treating each class equally and avoiding the dominance of majority classes in the final metric. This makes it more suitable for our scenario, where several specific edge prediction tasks involve rare or underrepresented classes. By using Balanced Accuracy, we ensure that the performance reported reflects how well the model generalizes across all classes, not just the frequent ones. On the other hand, for the specific-edge prediction tasks, which are binary tasks, in Supplementary Section S6 we also report the F1 scores.

S3 RNA-KG views

In this section, we provide a detailed description of the different views used for the predictive tasks.

Besides providing the biological motivation behind the construction of each view, we describe their topology by reporting the complementary cumulative distribution function for the node degrees with respect to the node types and the graph schema. Additionally, we describe the distribution of the node types and of the edge types.

In figure S2, we represent the complementary cumulative distribution function (CCDF) of all the RNA-KG views.

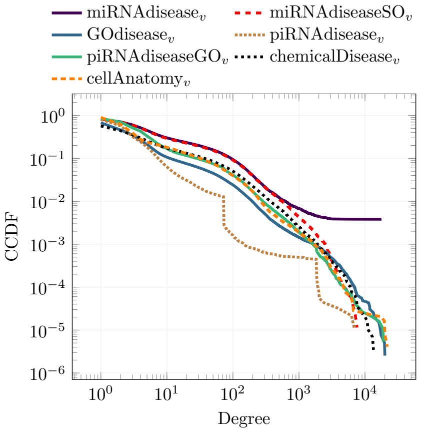

Figure S2: Complementary Cumulative Degree Distribution (CCDF) of RNA-KG views.

S3.1 miRNAdisease

The miRNAdisease view focuses on the interplay between miRNAs, genes, aberrant phenotypes, and diseases. miRNAs exhibit extremely high connectivity since they regulate hundreds of target genes and influence diverse disease [4]. Anatomical, chemical, and GO terms add context to the interactions involving miRNAs, genes, and diseases.

Figure S3: Left: Complementary Cumulative Distribution Function (CCDF) of the node degrees by node type for the miRNAdisease view. Right: Graph induced by the view types. In the graph, node size represent the proportion of nodes in the graph while edge width and color represent the proportion of edges between nodes of the respective types. In the figure we omitted node types accounting for less than 1% of the graph nodes.

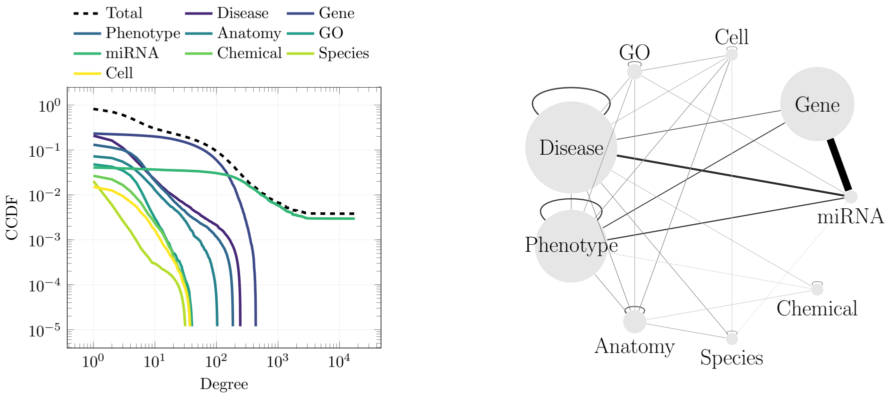

S3.2 miRNAdiseaseSO

The miRNAdiseaseSO view enriches the miRNAdisease view with genomic context. It introduces nodes of type Genomic feature stemming from the integration of the Sequence Ontology [3] according to the PheKnowLator ontology cleaning and integration strategy [2].

Table S2: Node distribution respect to the node type for the miRNAdisease view*.

Node	Count	Proportion	Mean degree
Disease	24203	0.2882	9.0892
Gene	19517	0.2324	71.7402
Phenotype	19152	0.2280	7.7664
Anatomy	6084	0.0724	8.6917
GO	4050	0.0482	5.9427
miRNA	3493	0.0416	423.4423
Chemical	2239	0.0267	5.6829
Species	1784	0.0212	3.1508
Cell	1272	0.0151	5.9041
Environment	453	0.0054	4.8234
Environmental exposure	419	0.0050	3.7613
Chromosome	310	0.0037	5.1839
Protein	205	0.0024	7.3366
Food	181	0.0022	2.8619
Neuro behaviour	153	0.0018	3.7647
Medical action	114	0.0014	3.7895
Genomic feature	98	0.0012	276.2857
NCI thesaurus	84	0.0010	3.1310

* We report only node types accounting for more than 0.1% of the graph nodes.

S3.3 GOdisease

The GOdisease view focuses on Gene Ontology [1] annotations for proteins, genes, and RNAs, mainly miRNAs and lncRNAs. This view facilitates the interpretation of disease mechanisms and potential pathogenic roles of non-coding RNAs across biological processes, molecular functions, and cellular components.

Table S3: Edge distribution respect to the type of the edge node extremes for the miRNAdisease view. We include the most frequent predicates for the extreme node types*.

**Source**	**Destination**	**Count**	**Proportion**	**Predicates**
miRNA	Gene	1333269	0.7869	Involved in regulation of
miRNA	Disease	105211	0.0621	Causes or contributes to condition
Disease	Disease	44117	0.0260	Subclassof (0.9444), Has characteristic (0.0367), Predisposes towards (0.0085)
Phenotype	Phenotype	38489	0.0227	Subclassof (25475/0.6619), Has modifier (0.1672), Has quality (0.1563)
miRNA	Phenotype	36752	0.0217	Causes or contributes to condition
Gene	Phenotype	24519	0.0145	Causes or contributes to condition
Gene	Genomic feature	23322	0.0138	Subclassof
Anatomy	Anatomy	20840	0.0123	Subclassof (0.5189), Part of (0.2536), Develops from (0.0452), Connects (0.0167), Has part (0.0139), Composed primarily of (0.0138), Contributes to morphology of (0.0118)
Gene	Disease	14422	0.0085	Causes or contributes to condition
GO	GO	10978	0.0065	Subclassof (0.7719), Part of (0.0580), Regulates (0.0575), Positively regulates (0.0505), Negatively regulates (0.0504)
Phenotype	Anatomy	6479	0.0038	Characteristic of (0.5309), Part of (0.2423), Characteristic of part of (0.1375)
Chemical	Chemical	5844	0.0034	Subclassof (0.5643), Has role (0.2033)
Disease	Gene	4622	0.0027	Has material basis in germline mutation in (0.9939)
miRNA	Genomic feature	3493	0.0021	Subclassof
Disease	Anatomy	2996	0.0018	Disease has location (0.8872), Disease has inflammation site (0.0844), Disease arises from alteration in structure (0.0197)
Cell	Cell	2380	0.0014	Subclassof (0.8924), Develops from (0.0983)
Species	Species	1782	0.0011	Subclassof
Disease	Phenotype	1655	0.0010	Disease has feature (0.4961), Has characteristic (0.3994), Disease has major feature (0.0550), Disease arises from feature (0.0387)

* We report only pair of node types accounting for more than 0.1% of the graph edges; and for each pair of node types, the predicates accounting for more than 1% of the edge set.

Figure S4: Left: Complementary Cumulative Distribution Function (CCDF) of the node degrees by node type for the miRNAdiseaseSO view. Right: Graph induced by the view types. In the graph, node size represent the proportion of nodes in the graph while edge width and color represent the proportion of edges between nodes of the respective types. In the figure we omitted node types accounting for less than 1% of the graph nodes.

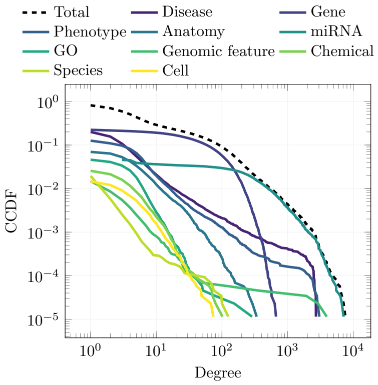

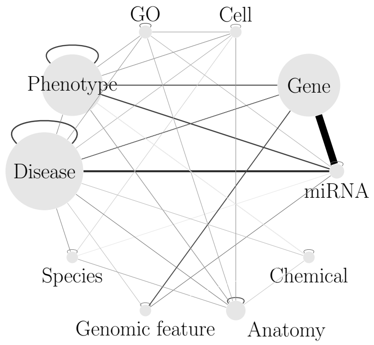

S3.4 piRNAdisease

The piRNAdisease view centers on associations between piRNAs and diseases/phenotypes. Given the emerging role of piRNAs in epigenetic regulation and genome stability [5], this view is integrated with anatomical, variant, SO (ge-

Table S4: Node distribution respect to the node type for the miRNAdiseaseSO view*.

Node	Count	Proportion	Mean degree
Disease	24203	0.2771	9.0895
Gene	19517	0.2234	71.7402
Phenotype	19152	0.2192	7.7664
Anatomy	6084	0.0696	8.6915
miRNA	4565	0.0523	326.7593
GO	4050	0.0464	5.9407
Genomic feature	2391	0.0274	14.3141
Chemical	2239	0.0256	5.6829
Species	1784	0.0204	3.1508
Cell	1272	0.0146	5.9041
Environment	453	0.0052	4.8256
Environmental exposure	419	0.0048	3.7613
Chromosome	310	0.0035	5.1839
Protein	205	0.0023	7.3366
Food	181	0.0021	2.8674
Neuro behaviour	153	0.0018	3.7647
Medical action	114	0.0013	3.7895
NCI thesaurus	84	0.0010	3.1190

* We report only node types accounting for more than 0.1% of the graph nodes.

nomic feature), and GO terms.

Table S5: Edge distribution respect to the type of the edge node extremes for the miRNAdiseasSO view. We include the most frequent predicates for the extreme node types*.

Source	Destination	Count	Proportion	Predicates
miRNA	Gene	1333269	0.7824	Involved in regulation of
miRNA	Disease	105211	0.0617	Causes or contributes to condition
Disease	Disease	44117	0.0259	Subclassof (0.9444), Has characteristic (0.0367)
Phenotype	Phenotype	38489	0.0226	Subclassof (0.6619), Has modifier (0.1672), Has quality (0.1563), Has part (0.0104), Increased in magnitude relative to (0.0014)
miRNA	Phenotype	36752	0.0216	Causes or contributes to condition
Gene	Phenotype	24519	0.0144	Causes or contributes to condition
Gene	Genomic feat.	23322	0.0137	Subclassof
Anatomy	Anatomy	20840	0.0122	Subclassof (0.5189), Part of (0.2491), Develops from (0.0457), Connects (0.0167), Contributes to morphology of (0.0143), Has part (0.0139), Composed primarily of (0.0138)
Gene	Disease	14422	0.0085	Causes or contributes to condition
GO	GO	10978	0.0064	Subclassof (0.7719), Part of (0.0580), Regulates (0.0575), Positively regulates (0.0505), Negatively regulates (0.0504)
Phenotype	Anatomy	6479	0.0038	Characteristic of (0.5314), Part of (0.2423), Characteristic of part of (0.1375), Occurs in (0.0585), Towards (0.0252)
Chemical	Chemical	5844	0.0034	Subclassof (0.5643), Has role (0.2033), Is conjugate acid of (0.0647), Is conjugate base of (0.0647), Has functional parent (0.0320), Is tautomer of (0.0250), Is enantiomer of (0.0192), Has part (0.0171)
miRNA	miRNA	5750	0.0034	Develops from (0.5000), Develops into (0.5000),
Disease	Gene	4622	0.0027	Has material basis in germline mutation in (0.9939)
miRNA	Genomic feat.	4565	0.0027	Subclassof
Genomic feat.	Genomic feat.	3145	0.0018	Subclassof (0.8003), Part of (0.0585), Has quality (0.0525), Has part (0.0483), Derives from (0.0207)
Disease	Anatomy	2996	0.0018	Disease has location (0.8868), Disease has inflammation site (0.0848), Disease arises from alteration in structure (0.0197)
Cell	Cell	2380	0.0014	Subclassof (0.8924), Develops from (0.0983)
Species	Species	1782	0.0010	Subclassof
Disease	Phenotype	1655	0.0010	Disease has feature (0.4967), Has characteristic (0.3994), Disease has major feature (0.0550), Disease arises from feature (0.0381)

* We report only pair of node types accounting for more than 0.1% of the graph edges; and for each pair of node types, the predicates accounting for more than 1% of the edge set.

Figure S5: Left: Complementary Cumulative Distribution Function (CCDF) of the node degrees by node type for the GOdisease view. Right: Graph induced by the view types. In the graph, node size represent the proportion of nodes in the graph while edge width and color represent the proportion of edges between nodes of the respective types. In the figure we omitted node types accounting for less than 1% of the graph nodes.

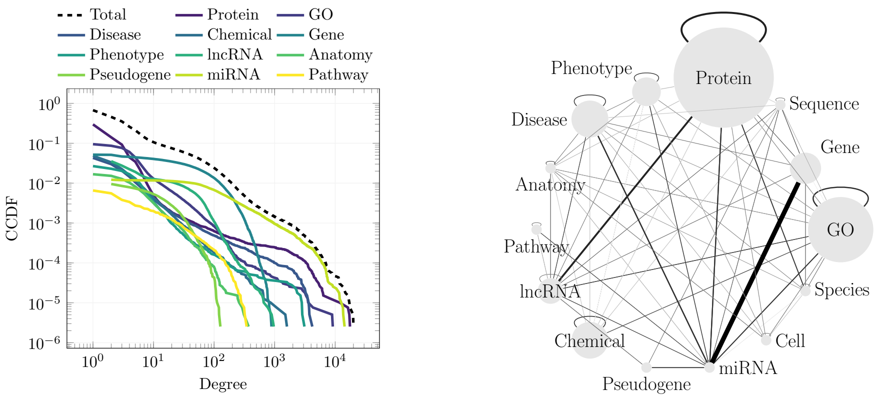

S3.5 piRNAdiseaseGO

The piRNAdiseaseGO view extends the piRNAdisease view by incorporating Gene Ontology (GO) annotations. This integration enables the functional characterization of piRNA molecules, providing insights into their potential roles in

Table S6: Node distribution respect to the node type for the miRNAdisease view*.

**Node**	**Count**	**Proportion**	**Mean degree**
Protein	217561	0.5524	4.0940
GO	42537	0.1080	9.6098
Disease	24073	0.0611	9.9476
Chemical	23922	0.0607	5.3946
Gene	20376	0.0517	74.0584
Phenotype	18713	0.0475	6.8853
lncRNA	16641	0.0422	19.0958
Anatomy	6597	0.0167	9.4050
Pseudogene	5156	0.0131	13.1389
miRNA	4800	0.0122	399.3890
Pathway	3850	0.0098	11.4460
Sequence	2386	0.0061	24.4778
Species	2352	0.0060	42.3899
Cell	1616	0.0041	6.5241
Glycan	797	0.0020	11.7465
Environment	446	0.0011	4.6502
Environmental exposure	419	0.0011	3.7542

* We report only node types accounting for more than 0.1% of the graph nodes.

Table S7: Edge distribution respect to the type of the edge node extremes for the GOdisease view. We include the most frequent predicates for the extreme node types*.

Source	Destination	Count	Proportion	Predicates
miRNA	Gene	1439378	0.4411	Regulates activity of (0.3451), Directly negatively regulates activity of (0.2432), Directly positively regulates activity of (0.2427), Indirectly negatively regulates activity of (0.1047), Indirectly positively regulates activity of (0.0643),
Protein	Protein	250975	0.0769	SubClass of (0.9958)
Protein	lncRNA	198287	0.0608	Interacts with
miRNA	Disease	113450	0.0348	Causes or contributes to condition
GO	GO	108502	0.0333	SubClass of (0.8038), Part of (0.0683), Regulates (0.0310), Positively regulates (0.0271), Negatively regulates (0.0269)
miRNA	Protein	81095	0.0249	Interacts with (0.9351), Involved in regulation of (0.0228), Is downstream of sequence of (0.0216), Is upstream of sequence of (0.0201)
Protein	miRNA	79244	0.0243	Interacts with (0.9569), Is upstream of sequence of (0.0221), Is downstream of sequence of (0.0206)
Protein	Species	65636	0.0201	Only in taxon
miRNA	Pseudogene	60011	0.0184	Regulates activity of
miRNA	GO	58472	0.0179	Participates in (0.7063), Has function (0.0874), Interacts with (0.0810), Involved in (0.0498), Located in (0.0428)
GO	miRNA	54571	0.0167	Has participant (0.7568), Function of (0.0937), Interacts with (0.0868), Location of (0.0459), Enabled by (0.0099)
Chemical	Chemical	49896	0.0153	SubClass of (0.7638), Has role (0.2101)
Disease	Disease	43545	0.0133	SubClass of (0.9435), Has characteristic (0.0371), Predisposes towards (0.0086)
miRNA	Phenotype	39220	0.0120	Causes or contributes to condition
Phenotype	Phenotype	37751	0.0116	SubClass of (0.6605), Has modifier (0.1679), Has quality (0.1567), Has part (0.0106)
lncRNA	GO	36476	0.0112	Obsolete contained in (0.7315), Ubiquitously expressed in (0.1131), Located in (0.0639), Under-expressed in (0.0583), Over-expressed in (0.0249)

* We report only pair of node types accounting for more than 0.1% of the graph edges; and for each pair of node types, the predicates accounting for more than 1% of the edge set.

Table S8: Node distribution respect to the node type for the piRNAdisease view*.

Node	Count	Proportion	Mean degree
Disease	24203	0.2624	6.6498
piRNA	23956	0.2597	5.3265
Phenotype	19152	0.2076	5.5463
Anatomy	6084	0.0660	8.6925
GO	4050	0.0439	5.9193
Gene	3823	0.0414	2.2090
Genomic feature	2391	0.0259	14.7633
Chemical	2239	0.0243	5.6833
Species	1784	0.0193	3.1485
Cell	1272	0.0138	5.9057
Variant (SNP)	1184	0.0128	23.8657
Environment	453	0.0049	4.8212
Environmental exposure	419	0.0045	3.7566
Chromosome	310	0.0034	5.1839
Protein	205	0.0022	7.3366
Food	181	0.0020	2.8619
Neuro behaviour	153	0.0017	3.7451
Medical action	114	0.0012	3.7895

* We report only node types accounting for more than 0.1% of the graph nodes.

Figure S6: Left: Complementary Cumulative Distribution Function (CCDF) of the node degrees by node type for the miRNAdisease view. Right: Graph induced by the view types. In the graph, node size represent the proportion of nodes in the graph while edge width and color represent the proportion of edges between nodes of the respective types. In the figure we omitted node types accounting for less than 1% of the graph nodes.

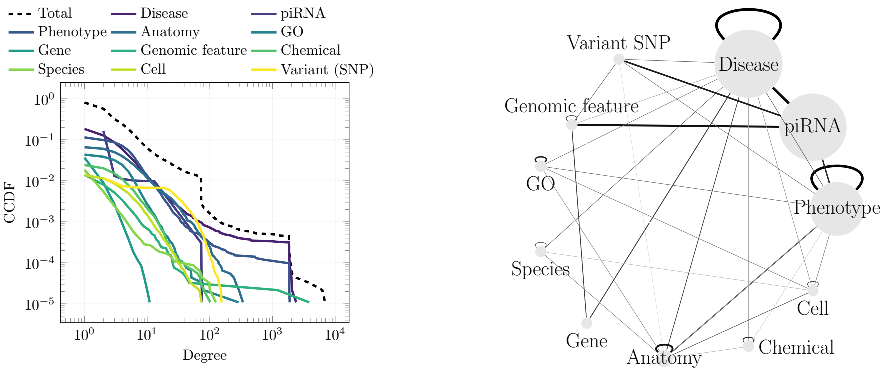

biological processes, molecular functions, and cellular components.

Figure S7: Left: Complementary Cumulative Distribution Function (CCDF) of the node degrees by node type for the miRNAdisease view. Right: Graph induced by the view types. In the graph, node size represent the proportion of nodes in the graph while edge width and color represent the proportion of edges between nodes of the respective types. In the figure we omitted node types accounting for less than 0.3% of the graph nodes.

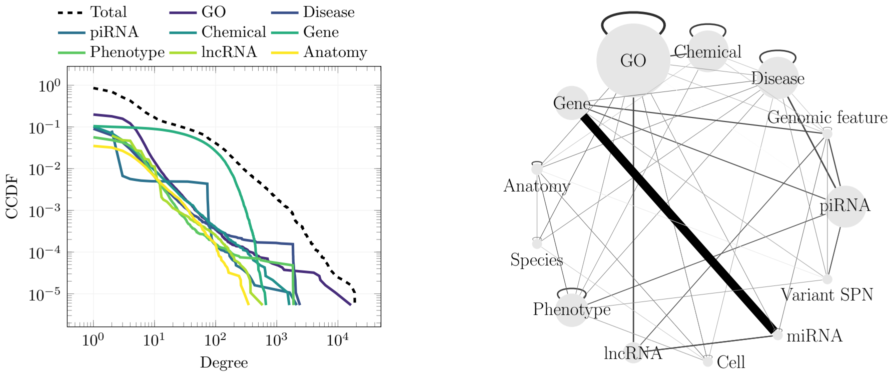

S3.6 chemicalDisease

The chemicalDisease view focuses on the relationships between chemicals, diseases, genes, phenotypes, and non-coding RNAs. This view is particularly relevant for exploring how chemical exposures affect disease development. The view is suited for prioritizing chemical-disease associations and identifying candidate miRNAs and lncRNAs involved in these interactions.

Table S9: Edge distribution respect to the type of the edge node extremes for the piRNAdisease view. We include the most frequent predicates for the extreme node types*.

**Source**	**Destination**	**Count**	**Proportion**	**Predicates**
Disease	Disease	44117	0.1521	Subclassof (0.9444), Has characteristic (0.0367), Predisposes towards (0.0085)
Phenotype	Phenotype	38489	0.1327	Subclassof (0.6619), Has modifier (0.1672), Has quality (0.1563), Has part (0.0104)
piRNA	Disease	35972	0.1240	Causally influenced by (0.6599), Causes or contributes to condition (0.3401),
piRNA	Genomic feature	23956	0.0826	Subclassof
Disease	piRNA	23738	0.0819	Causally influences
Anatomy	Anatomy	20840	0.0719	Subclassof (0.5189), Part of (0.2486), Develops from (0.0456), Connects (0.0167), Contributes to morphology of (0.0146), Has part (0.0139), Composed primarily of (0.0137)
piRNA	Variant (SNP)	12838	0.0443	Causally influenced by
GO	GO	10978	0.0379	Subclassof (0.7719), Part of (0.0580), Regulates (0.0575), Positively regulates (0.0505), Negatively regulates (0.0504)
piRNA	Phenotype	9130	0.0315	Causally influenced by
Phenotype	piRNA	9130	0.0315	Causally influences
Phenotype	Anatomy	6479	0.0223	Characteristic of (0.5348), Part of (0.2423), Characteristic of part of (0.1375), Occurs in (0.0585), Towards (0.0218)
Chemical	Chemical	5844	0.0202	Subclassof (0.5643), Has role (0.2033), Is conjugate acid of (0.0647), Is conjugate base of (0.0647), Has functional parent (0.0320), Is tautomer of (0.0250), Is enantiomer of (0.0192), Has part (0.0171)
Disease	Gene	4622	0.0159	Has material basis in germline mutation in (0.9929)
Gene	Genomic feature	3823	0.0132	Subclassof
Genomic feature	Genomic feature	3145	0.0108	Subclassof (0.8003), Part of (0.0585), Has quality (0.0525), Has part (0.0483), Derives from (0.0207)
Disease	Anatomy	2996	0.0103	Disease has location (0.8879), Disease has inflammation site (0.0841), Disease arises from alteration in structure (0.0194)
Cell	Cell	2380	0.0082	Subclassof (0.8924), Develops from (0.0983)
Species	Species	1782	0.0061	Subclassof
Disease	Phenotype	1655	0.0057	Disease has feature (0.4961), Has characteristic (0.3994), Disease has major feature (0.0550), Disease arises from feature (0.0387)
Disease	Species	1435	0.0049	In taxon (0.4718), Disease has infectious agent (0.3171), Disease has primary infectious agent (0.0899), Subclassof (0.0669), Transmitted by (0.0523)
Variant (SNP)	Genomic feature	1184	0.0041	Subclassof
Environment	Environment	924	0.0032	Subclassof (0.5260), Part of (0.1082), Composed primarily of (0.0920), Has part (0.0476), Has participant (0.0303), Characteristic of (0.0271), Overlaps (0.0227), Adjacent to (0.0195), Determined by (0.0173), Has output (0.0173), Has input (0.0130), Derives from (0.0119)
Variant (SNP)	Disease	880	0.0030	Causes or contributes to condition

* We report only pair of node types accounting for more than 0.3% of the graph edges; and for each pair of node types, the predicates accounting for more than 1% of the edge set.

Figure S8: Left: Complementary Cumulative Distribution Function (CCDF) of the node degrees by node type for the chemicalDisease view. Right: Graph induced by the view types. In the graph, node size represent the proportion of nodes in the graph while edge width and color represent the proportion of edges between nodes of the respective types. In the figure we omitted node types accounting for less than 0.4% of the graph nodes.

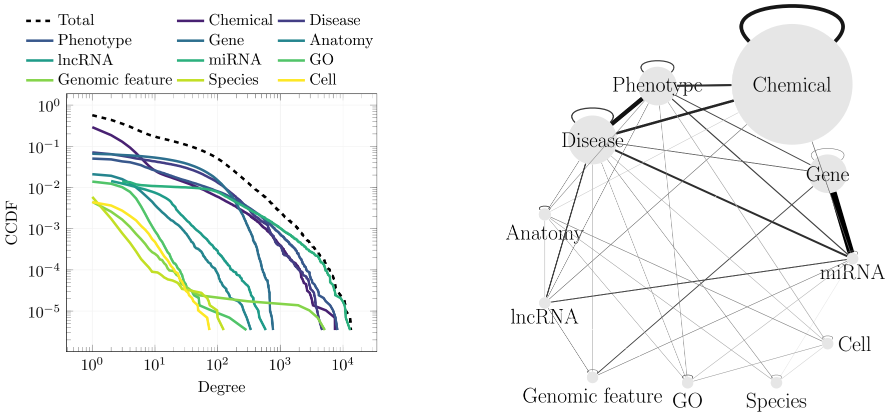

Table S10: Node distribution respect to the node type for the piRNAdiseaseGO view*.

Node	Count	Proportion	Mean degree
GO	42385	0.2265	7.3411
Disease	24203	0.1293	6.6499
piRNA	24048	0.1285	6.0304
Chemical	23964	0.1281	5.4079
Gene	19591	0.1047	70.3670
Phenotype	19168	0.1024	5.5539
lncRNA	12105	0.0647	7.4527
Anatomy	6561	0.0351	8.8113
miRNA	4565	0.0244	300.6697
Genomic feature	2391	0.0128	29.9950
Species	2370	0.0127	6.0025
Cell	1617	0.0086	6.4917
Variant (SNP)	1186	0.0063	23.9553
Protein	563	0.0030	5.5240
Environment	453	0.0024	4.8234
Environmental exposure	419	0.0022	3.7637
Chromosome	310	0.0017	5.1839
Plant	267	0.0014	4.5543
Food	181	0.0010	2.8674

* We report only node types accounting for more than 0.1% of the graph nodes.

S3.7 cellAnatomy

The cellAnatomy view emphasizes the functional location of biological entities across cellular and anatomical levels. This view includes interactions between cells, anatomical entities, and proteins, RNAs, and genes. It is particularly useful for studying cell-specific gene expression, tissue-specific regulation, and predicting the anatomical context of bio-molecules.

Table S11: Edge distribution respect to the type of the edge node extremes for the piRNAdiseaseGO view. We include the most frequent predicates for the extreme node types*.

**Source**	**Destination**	**Count**	**Proportion**	**Predicates**
miRNA	Gene	1333269	0.6771	Regulates activity of
GO	GO	107572	0.0546	Subclassof (0.8036), Part of (0.0681), Regulates (0.0310), Positively regulates (0.0272), Negatively regulates (0.0270)
Chemical	Chemical	50158	0.0255	Subclassof (0.7605), Has role (0.2094), Is conjugate acid of (0.0075), Is conjugate base of (0.0075)
Disease	Disease	44117	0.0224	Subclassof (0.9444), Has characteristic (0.0367), Predisposes towards (0.0085)
Phenotype	Phenotype	38497	0.0196	Subclassof (0.6619), Has modifier (0.1672), Has quality (0.1563), Has part (0.0104)
piRNA	Disease	35972	0.0183	Causally influenced by (0.6599), Causes or contributes to condition (0.3401),
GO	Chemical	28008	0.0142	Has participant (0.7509), Has primary input (0.1467), Has primary output (0.0496), Has primary input or output (0.0445)
lncRNA	GO	27785	0.0141	Located in (0.9962), Involved in (0.0038),
GO	lncRNA	27680	0.0141	Location of
piRNA	Genomic feature	24048	0.0122	Subclassof
Disease	piRNA	23738	0.0121	Causally influences
Gene	Genomic feature	23364	0.0119	Subclassof
miRNA	lncRNA	22644	0.0115	Interacts with
lncRNA	miRNA	22644	0.0115	Interacts with
Anatomy	Anatomy	22502	0.0114	Subclassof (0.5243), Part of (0.2499), Develops from (0.0444), Connects (0.0158), Contributes to morphology of (0.0154), Has part (0.0134), Composed primarily of (0.0129)
piRNA	Gene	17152	0.0087	Regulates activity of
piRNA	Variant (SNP)	12838	0.0065	Causally influenced by
Variant (SNP)	piRNA	12838	0.0065	Causally influences
lncRNA	Genomic feature	12105	0.0061	Subclassof
piRNA	Phenotype	9214	0.0047	Causally influenced by
Phenotype	piRNA	9214	0.0047	Causally influences
Phenotype	Anatomy	6479	0.0033	Characteristic of (0.5305), Part of (0.2423), Characteristic of part of (0.1375), Occurs in (0.0585), Towards (0.0261)
GO	Species	6259	0.0032	In taxon (0.6148), Subclassof (0.3189), Only in taxon (0.0633)
miRNA	miRNA	5750	0.0029	Develops from (0.5000), Develops into (0.5000),
Disease	Gene	4622	0.0023	Has material basis in germline mutation in (0.9924), Disease has basis in dysfunction of (0.0035)
miRNA	Genomic feature	4565	0.0023	Subclassof
Species	Species	3417	0.0017	Subclassof (0.7038), In taxon (0.2962),
Genomic feature	Genomic feature	3145	0.0016	Subclassof (0.8003), Part of (0.0588), Has quality (0.0525), Has part (0.0483), Derives from (0.0207)
Cell	Cell	3005	0.0015	Subclassof (0.8769), Develops from (0.1105)
Disease	Anatomy	2996	0.0015	Disease has location (0.8868), Disease has inflammation site (0.0851), Disease arises from alteration in structure (0.0194)

* We report only pair of node types accounting for more than 0.1% of the graph edges; and for each pair of node types, the predicates accounting for more than 1% of the edge set.

Figure S9: Left: Complementary Cumulative Distribution Function (CCDF) of the node degrees by node type for the cellAnatomy view. Right: Graph induced by the view types. In the graph, node size represent the proportion of nodes in the graph while edge width and color represent the proportion of edges between nodes of the respective types. In the figure we omitted node types accounting for less than 0.35% of the graph nodes.

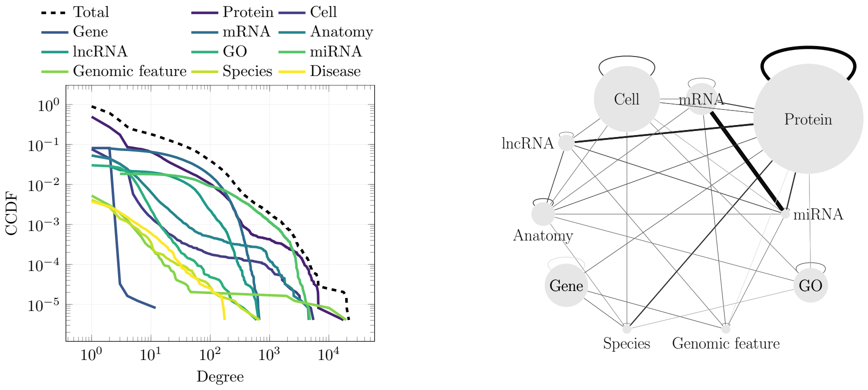

S4 Node Type Prediction

The node-type prediction task is a multi-class classification problem aimed at determining the type of a node among the N most frequently occurring node types. In this setting, the number of classes is $N+1$, where the $(N+1)$-th class includes all node types that do not belong to the N most represented categories.

For example, if $N = 7$, the seven most common node types in RNA-KG

Table S12: Node distribution respect to the node type for the chemicalDisease view*.

Node	Count	Proportion	Mean degree
Chemical	199178	0.6893	6.2203
Disease	24203	0.0838	61.0638
Phenotype	19152	0.0663	65.7421
Gene	19075	0.0660	67.3030
Anatomy	6084	0.0211	8.6995
lncRNA	5123	0.0177	15.5257
miRNA	4565	0.0158	306.4458
GO	4050	0.0140	5.9437
Genomic feature	2391	0.0083	16.2685
Species	1784	0.0062	3.1508
Cell	1272	0.0044	5.9033
Environment	453	0.0016	4.8256
Environmental exposure	419	0.0014	3.7589
Chromosome	310	0.0011	5.1839

* We report only node types accounting for more than 0.1% of the graph nodes.

(i.e. Chemical, Protein, GO, Cell line, Disease, tRF, and mRNA) constitute the primary classes in the multi-class classification problem, while the 8th class encompasses all other node types.

For this task, node embeddings were generated using BFS-like Node2Vec with a skip-gram model (using GRAPE's default hyperparameters). We computed embeddings of the following dimensions: 100-dimensional (100-D), 50-D, 10-D, and 2-D. Note that the 2-D embeddings were obtained by first generating 100-D embeddings and subsequently reducing their dimensionality with t-SNE.

These embeddings were then used in three consecutive experiments, where we trained and tested three classification models: decision trees (DTs), random

Table S13: Edge distribution respect to the type of the edge node extremes for the chemicalDisease view. We include the most frequent predicates for the extreme node types*.

**Source**	**Destination**	**Count**	**Proportion**	**Predicates**
miRNA	Gene	1213775	0.3497	Regulates activity of (0.3696), Directly negatively regulates activity of (0.2426), Directly positively regulates activity of (0.2421) , Indirectly negatively regulates activity of (0.0898), Indirectly positively regulates activity of (0.0558),
Disease	Phenotype	456307	0.1315	Has phenotype (0.9964), Disease has feature (0.0018)
Phenotype	Disease	454652	0.1310	Phenotype of
Chemical	Chemical	368859	0.1063	Subclassof (0.7501), Has role (0.1185), Has functional parent (0.0533), Is conjugate acid of (0.0231), Is conjugate base of (0.0231), Has part (0.0109)
Disease	Chemical	151068	0.0435	Is treated by substance (0.9997)
Chemical	Disease	151019	0.0435	Is substance that treats
miRNA	Disease	105211	0.0303	Causes or contributes to condition
Phenotype	Chemical	99295	0.0286	Is treated by substance (0.9931), Characteristic of (0.0066)
Chemical	Phenotype	98613	0.0284	Is substance that treats Subclassof (0.0000),
lncRNA	Disease	46603	0.0134	Causes or contributes to condition
Disease	Disease	44117	0.0127	Subclassof (0.9444)
Phenotype	Phenotype	38489	0.0111	Subclassof (0.6619), Has modifier (0.1672), Has quality (0.1563), Has part (0.0104)
miRNA	Phenotype	36752	0.0106	Causes or contributes to condition
Gene	Phenotype	24519	0.0071	Causes or contributes to condition
Gene	Genomic feature	22872	0.0066	Subclassof
lncRNA	miRNA	22644	0.0065	Interacts with
miRNA	lncRNA	22644	0.0065	Interacts with
Anatomy	Anatomy	20840	0.0060	Subclassof (0.5189), Part of (0.2517), Develops from (0.0453), Connects (0.0166), Has part (0.0139), Composed primarily of (0.0138), Contributes to morphology of (0.0122)
Gene	Disease	14422	0.0042	Causes or contributes to condition
GO	GO	10978	0.0032	Subclassof (0.7719), Part of (0.0580), Regulates (0.0575), Positively regulates (0.0505), Negatively regulates (0.0504)
Phenotype	Anatomy	6479	0.0019	Characteristic of (0.5309), Part of (0.2423), Characteristic of part of (0.1375), Occurs in (0.0585), Towards (0.0256)
miRNA	miRNA	5750	0.0017	Develops from (0.5000), Develops into (0.5000),
lncRNA	Genomic feature	5123	0.0015	Subclassof
Disease	Gene	4622	0.0013	Has material basis in germline mutation in (0.9937), Disease has basis in dysfunction of (0.0022)
miRNA	Genomic feature	4565	0.0013	Subclassof
Chemical	miRNA	4119	0.0012	Interacts with
miRNA	Chemical	4119	0.0012	Interacts with
lncRNA	Phenotype	3821	0.0011	Causes or contributes to condition
Gene	Gene	3594	0.0010	Genetically interacts with

* We report only pair of node types accounting for more than 0.1% of the graph edges; and for each pair of node types, the predicates accounting for more than 1% of the edge set.

forests (RFs), and radial basis function support vector machines (RBF-SVMs). Hyperparameter optimization for all models was performed via 5-fold stratified grid-search cross-validation on t he training set.

Our experiments began with the 2-D embeddings and by testing with $N = \{7, 20, 54\}$.

Supplementary Tables S16, S17, and S18 detail the hyperparameters used for model selection of DTs, RFs, and RBF-SVMs when trained on these t-SNE-reduced embeddings. Results achieved in this experiments are shown in supplementary figure S10. RBF-SVMs achieved performance comparable to RFs but required significantly more time to converge. To mitigate this issue, we optimized the RBF-SVM hyperparameters using a subset of the training graph rather than the full dataset. However, the slower convergence and similar performance compared to RFs ultimately led us to *exclude RBF-SVMs from further experiments, focusing instead on RFs and DTs.* We also noticed that, as expected, increasing the number of considered node-types (classes) has a significant impact on performance. This behavior can be explained by the fact that not all node types are as easily separable from the others; this is suggested by their 2D embeddings which tend to overlap (see the 2D t-SNE node embeddings of a subset of less represented RNA-related types in Supplementary Figure S11).

In the second experiment, we optimized DT hyperparameters and compared the results across all embedding sizes by testing with $N = \{7, 20, 54, 68, 81\}$. Hyperparameter optimization was performed using the values listed in Supplementary Table S19. The performance obtained with different embedding sizes is illustrated in Supplementary Figure S12.

Observing the results, we determined that 10-D embeddings performed comparably to, or slightly better than, the 50-D and 100-D embeddings. This finding led us to continue our experiments using 10-D embeddings. The detailed results for the 10-D embedding size are reported in the main paper, while Tables S20

Table S14: Node distribution respect to the node type for the miRNAdisease view*.

**Node**	**Count**	**Proportion**	**Mean degree**
Protein	122682	0.4991	12.2548
Cell	40516	0.1648	4.0871
Gene	20009	0.0814	3.0028
mRNA	19613	0.0798	63.9203
Anatomy	14488	0.0589	12.6432
lncRNA	10100	0.0411	28.9183
GO	7427	0.0302	6.9841
miRNA	4565	0.0186	281.6981
Genomic feature	2391	0.0097	25.3371
Species	1064	0.0043	114.5385
Disease	926	0.0038	7.8197
Chemical	803	0.0033	5.6961
Glycan	797	0.0032	11.7465

* We report only node types accounting for more than 0.1% of the graph nodes.

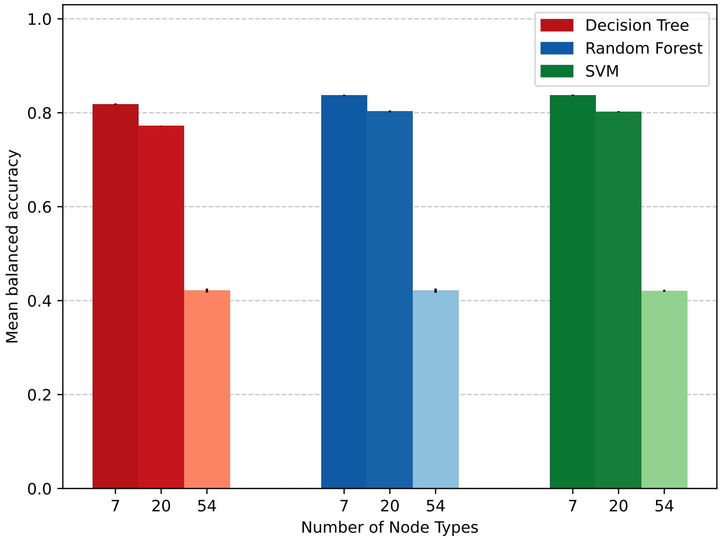

Figure S10: Balanced accuracy results on RNA-KG node type prediction using 2-D embeddings of BFS-like Node2Vec embeddings and DTs (red bars), RFs (blue bars), and RBF-SVM (green bars) as classifier models. Classification results are relative to the 7, 20 and 54 most common node types. The color intensity of the bars reflects their balanced accuracy value. Vertical segments on each point represent the standard deviation across multiple holdouts; note that in some cases the standard deviation is very low ($< 0.1\%$) and the segment is not visible.

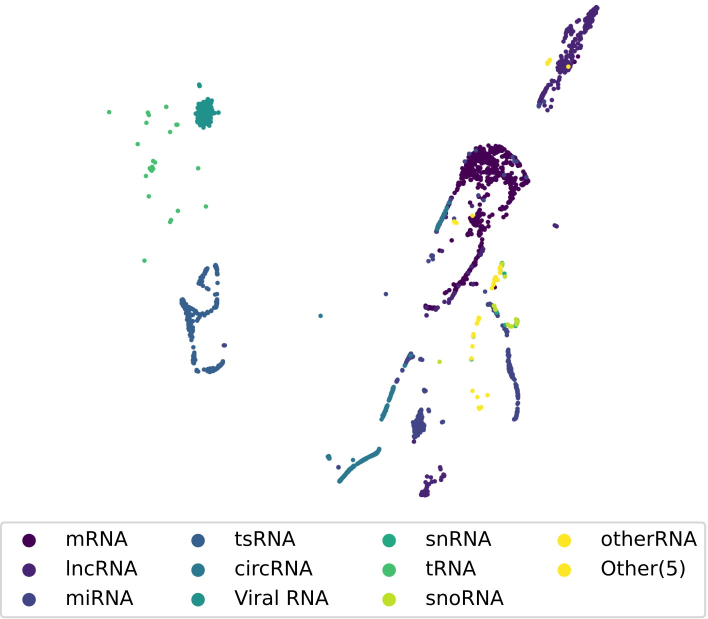

Figure S11: 2D t-SNE projections of node embeddings of the RNA-KG showing only RNA-related node types.

and S21 present the results for the 50-D and 100-D embeddings, respectively. The hyperparameters selected for different embedding sizes are as follows:

- **DT, 2-D embeddings**: Maximum tree depth = 20.

Table S15: Edge distribution respect to the type of the edge node extremes for the cellAnatomy view. We include the most frequent predicates for the extreme node types*.

**Source**	**Destination**	**Count**	**Proportion**	**Predicates**
miRNA	mRNA	1158937	0.3098	Directly regulates activity of (0.5005), Interacts with (0.4994), Regulates activity of (0.0001),
Protein	Protein	784051	0.2096	Molecularly interacts with (0.7893), Subclassof (0.2093)
mRNA	miRNA	578817	0.1547	Interacts with
Protein	lncRNA	200567	0.0536	Interacts with
Protein	Species	91501	0.0245	Only in taxon
miRNA	Protein	82001	0.0219	Interacts with (0.9361), Involved in regulation of (0.0226), Is downstream of sequence of (0.0214), Is upstream of sequence of (0.0199),
Protein	miRNA	80150	0.0214	Interacts with (0.9578), Is upstream of sequence of (0.0219), Is downstream of sequence of (0.0204),
Cell	Cell	58487	0.0156	Subclassof (0.8577), Derives from (0.1091), Is in cell line repository (0.0279)
mRNA	Protein	45615	0.0122	Interacts with (0.5840), Ribosomally translates to (0.4160),
Protein	mRNA	45615	0.0122	Interacts with (0.5840), Ribosomal translation of (0.4160),
Anatomy	Anatomy	42990	0.0115	Subclassof (0.5582), Part of (0.2571), Develops from (0.0351), Connects (0.0129), Composed primarily of (0.0117)
Protein	Protein modification	32450	0.0087	Has part (0.9999), Subclassof (0.0001),
lncRNA	Anatomy	25679	0.0069	Located in
Anatomy	lncRNA	25679	0.0069	Location of
GO	GO	23719	0.0063	Subclassof (0.7456), Part of (0.0939), Regulates (0.0470), Negatively regulates (0.0407), Positively regulates (0.0404)
lncRNA	miRNA	22644	0.0061	Interacts with
miRNA	lncRNA	22644	0.0061	Interacts with
Protein	Gene	20088	0.0054	Has gene template
Gene	Genomic feature	19976	0.0053	Subclassof
Gene	Species	19975	0.0053	Only in taxon
mRNA	Genomic feature	19613	0.0052	Subclassof
Anatomy	Protein	16214	0.0043	Location of (0.9907), Subclassof (0.0085), Has part (0.0004), Produces (0.0003), Composed primarily of (0.0001),
Protein	Anatomy	16064	0.0043	Located in
lncRNA	Genomic feature	10100	0.0027	Subclassof
Cell	Protein	9862	0.0026	Location of (0.9597), Has plasma membrane part (0.0177), Lacks plasma membrane part (0.0122), Subclassof (0.0044)
Protein	Cell	9473	0.0025	Located in (0.9992), Part of (0.0008),
Protein	Glycan	7769	0.0021	Has part
miRNA	miRNA	7460	0.0020	Develops from (0.3854), Develops into (0.3854), In homology relationship with (0.1147), Genetically interacts with (0.1123)
mRNA	mRNA	7034	0.0019	Interacts with
Cell	Species	6914	0.0018	Part of (0.9954), Only in taxon (0.0022)
Cell	Anatomy	6746	0.0018	Part of (0.9050), Subclassof (0.0434), Derives from (0.0237), Existence starts during (0.0120), Has soma location (0.0099)
Cell	Experimental factor	4731	0.0013	Is disease model for (0.9979), Part of (
miRNA	Genomic feature	4565	0.0012	Subclassof
Cell	Disease	4318	0.0012	Is disease model for (0.7791), Derives from patient having disease (0.1202), Has disease (0.1003)

* We report only pair of node types accounting for more than 0.1% of the graph edges; and for each pair of node types, the predicates accounting for more than 1% of the edge set.

Parameter	Values
Tree depth	5, 10, 12, 15, 20, 25, 30, 40, 50

Table S16: Hyperparameters and values used for model selection of DTs for node type prediction based on the 2D t-SNE projections of the embeddings.

Parameter	Values
Tree depth	20
Number of trees	1, 10, 50, 100, 200, 500

Table S17: Hyperparameters and values used for model selection of RFs for node type prediction based on the 2D t-SNE projections of the embeddings.

- **DT, 10-D embeddings**: Maximum tree depth = 50, maximum features = 5.
- **DT, 50-D embeddings**: Maximum tree depth = 40, maximum features = 20.
- **DT, 100-D embeddings**: Maximum tree depth = 40, maximum features = 100.

The final experiment focused on RF hyperparameter optimization with a fixed embedding size of 10-D. We then compared the results across different embedding sizes by testing with $N = \{7, 20, 54, 68, 81\}$.

Supplementary Table S22 details the hyperparameters used for model selection of RFs trained on the full 10-D embeddings generated with BFS-like Node2Vec Skip-Gram. The optimized hyperparameters used for the RF results reported are as follows:

- **RF, 2-D embeddings**: Maximum tree depth = 20, number of trees = 500.
- **RF, 10-D embeddings**: Maximum features = 5, number of trees = 300.

S5 Generic Edge Prediction

The *generic* edge prediction task aims to predict the existence of edges independently of their type. To esnure an unbiased evaluation, we applied a 5-fold stratified cross-validation (70:30, train:test) procedure. Connected Monte-Carlo holdouts were leveraged to select the positive edges of the training set using a spanning tree. The same random seed generator was used to ensure comparability of predictive results. Nodes in the training set were embedded using a BFS-like node2vec approach with Skip-Gram model, employing the default

Parameter	Values
C	1.0e-02, 1.0e+00, 1.0e+01, 3.2e+02, 1.0e+04, 1.0e+05, 1.0e+07, 3.2e+07, 1.0e+10]
γ	1.0e-09, 1.0e-06, 1.0e-03, 1.0e+00, 5.6e+00, 3.2e+01, 1.8e+02, 1.0e+03

Table S18: Hyperparameters and values used for model selection of RBF-SVMs for node type prediction based on the 2D t-SNE projections of the embeddings.

Parameter	Values
Tree depth	5, 10, 15, 20, 25, 30, 40, 50, 60, 70, 80
Max features	1, 'sqrt', 5, 7, 9, 10,20,35,40,50,60,80,100

Table S19: Hyperparameters and values used for model selection of DTs for node type prediction on 10-D, 50-D, and 100-D embeddings.

GRAPE hyperparameters and varying embedding sizes (i.e. 10-D, 50-D, and 100-D). The edge embeddings are generated by concatenating the embeddings of the source and destination nodes. Finally, node-degree aware sampling was used to randomly generate negative edges for the training and test sets. Supervised classification was performed using DT and RF models, whose hyperparameters were optimized exploiting a 5-fold stratified grid-search cross-validation on the training set. We considered tree depth, maximum features and number of trees for tuning. Table S23 shows the hyperparameters combinations tested with DT predictors. The number of trees for RF was tuned considering 10, 50, 100, 200 and 500 trees. The other main parameters (tree depth and maximum number of features) were set as the best performing hyperparameters combination found during the DT tuning for each embedding size.

The hyperparameters used to obtain the results shown in the paper for generic edge prediction task are as follows:

- **DT, 10-D embeddings**: 50 maximum tree depth and 10 maximum features.
- **DT, 50-D embeddings**: 200 maximum tree depth and 50 maximum features.
- **DT, 100-D embeddings**: 50 maximum tree depth and 50 maximum features.
- **RF, 10-D embeddings**: 50 maximum tree depth, 10 maximum features, 200 trees.
- **RF, 50-D embeddings**: 200 maximum tree depth, 10 maximum features, 500 trees.

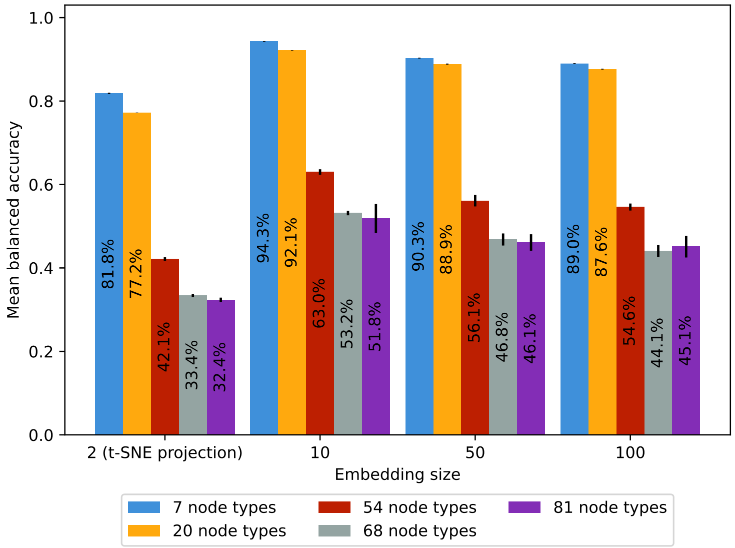

Figure S12: Bar plot comparing the performance of the DT model for node type prediction on the full RNA-KG considering different embedding sizes.

#Classes	Balanced Accuracy	Time (s)
7	90.29% ± 0.09%	162.420612
20	88.85% ± 0.12%	180.897616
54	56.12% ± 1.37%	331.611162
68	46.82% ± 1.45%	428.399223
81	46.10% ± 1.97%	449.408932

Table S20: Results obtained with the DT model for node type prediction based on the 50-D embedding with various numbers of node types to predict.

- **RF, 100-D embeddings**: 50 maximum tree depth, 50 maximum features, 500 trees.

S6 Specific Edge Prediction

The goal of the *specific* edge prediction task is to determine the existence of edges constrained to specific types of source and destination nodes. This task closely aligns with real-world applications, where researchers are often interested in identifying specific novel connections between entities.

To ensure a fair evaluation, we leveraged the unbiased pipeline proposed in Section 3.3 (implementation details in Supplementary Section S7) to construct positive and negative edges for the training and test sets, consider-

#Classes	Balanced Accuracy	Time (s)
7	88.99% ± 0.10%	857.78
20	87.64% ± 0.09%	919.63
54	54.60% ± 0.84%	1185.85
68	44.07% ± 1.41%	1303.05
81	45.11% ± 2.60%	1339.91

Table S21: Results obtained with the DT model for node type prediction based on the 100-D embedding with various numbers of node types to predict.

Parameter	Values
Max features	3, 5, 7, 10
Number of trees	50, 100, 150, 200, 250, 300

Table S22: Parameters and values used for model selection of RFs for node type prediction based on the full 10-dimensional embeddings.

Max features	Tree depth
'sqrt'	10
'sqrt'	50
'sqrt'	200
'sqrt'	500
25	50
25	200
25	500
50	50
50	200
50	500

Table S23: Hyperparameters evaluated for the selection of DT models in generic edge type prediction.

ing specific views of RNA-KG (i.e., miRNA-Disease, miRNA-DiseaseSO, GO-Disease, piRNA-Disease, piRNA-DiseaseGO, Chemical-Disease, Cell Anatomy views). Further details about RNA-KG views are available at `https://rna-kg.anacleto.di.unimi.it/views/`.

Figure S13 shows preliminary results to compare First-order LINE and two different parametrization for the Node2Vec Skip-Gram embedding (BFS-like and DFS-like embeddings). The preliminary results showed a slight preference towards the BFS-like parametrization for Node2Vec, which motivates its usage in subsequent experiments.

First-order LINE and BFS-like Node2Vec with Skip-gram model were used to compute the embeddings (size 10-D), which serves as input for supervised

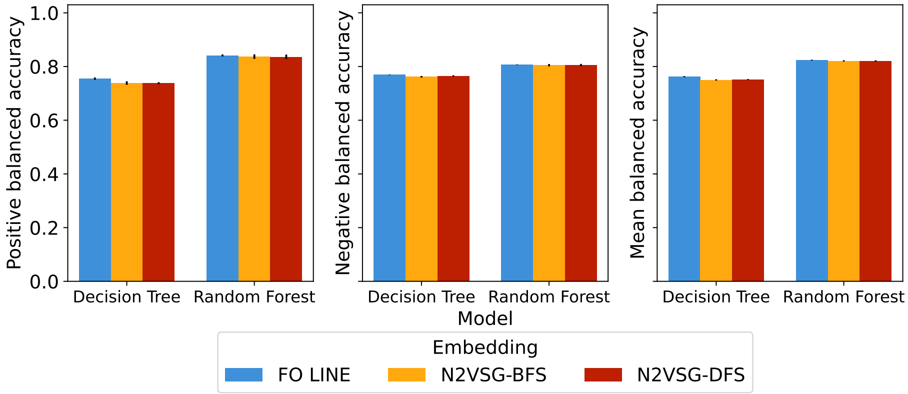

Figure S13: Bar plots comparing the preliminary performance of different embeddings with DT and RF models for miRNA-Disease specific edge prediction on miRNA-Disease view.

classification via DT and RF models. A 5-fold stratified cross-validation (70:30, train:test) was employed to evaluate the generalization performance. The same random seed generator was used to ensure comparability of predictive results.

The hyperparameters used to obtain the results for specific edge prediction are:

- DT: 100 maximum tree depth, 'sqrt' maximum features.
- RF: 100 maximum tree depth and 'sqrt' maximum features and 100 trees.

The following sections report the predictive performance on the RNA-KG views for different edge-types (we recall that the edge type is characterized by the types of its vertexes). We run each specific-edge type prediction task by using two different Unbalance Rates (UR). When UR = 1, the test set is balanced, i.e. it consists of an equal number of existing and not-existing edges; when UR = 10, the test set contains ten times as many not-existing edges as existing ones.

Besides the Balanced Accuracies, we report the F1 score and the weighted F1 score. The F1 score is computed by considering the existent edges as the positive class, and then calculating the harmonic mean of precision and recall:

$$F1 = \frac{2TP}{2TP + FP + FN}$$

being TP the number of true positive edges, FP the number of false positive edges, and FN the number of false negative edges. The weighted F1 score (F1-Weighted) is the weighted average of the F1 scores computed for each class independently (i.e. by using the existing edges as the positive class and by using the negative edges as the positive class in the calculation of the F1), where the weights are defined as the proportion of the number of true instances (support) for each class.

S6.1 MiRNAdisease View

The Balanced Accuracy (BA) results for the specific edge prediction task on the type pairs are reported in table S24 for the LINE embeddings and table S25 for the Node2Vec embeddings.

Table S24: Results of the DT and RF models for specific edge prediction with 10 dimensions embeddings obtained from LINE on miRNAdisease$_v$.

Model	Type pair	UR	Pos BA(%)	Neg BA(%)	Overall BA(%)	F1(%)	F1-Weighted(%)
DecisionTree	miRNA-Disease	1	75.98±0.44	74.34±0.42	75.16±0.24	75.36±0.26	75.16±0.24
DecisionTree	miRNA-Disease	10	75.98±0.44	81.80±0.11	78.89±0.21	42.45±0.20	84.60±0.07
DecisionTree	miRNA-Gene	1	59.36±0.14	57.81±0.11	58.59±0.11	58.91±0.12	58.58±0.11
DecisionTree	miRNA-Gene	10	59.36±0.14	61.82±0.09	60.59±0.11	21.94±0.08	69.75±0.07
DecisionTree	miRNA-Phenotype	1	77.01±0.40	75.36±0.18	76.19±0.16	76.38±0.21	76.18±0.16
DecisionTree	miRNA-Phenotype	10	77.01±0.40	85.67±0.28	81.34±0.27	48.09±0.50	87.24±0.20
RandomForest	miRNA-Disease	1	84.29±0.33	78.71±0.44	81.50±0.15	82.00±0.13	81.49±0.15
RandomForest	miRNA-Disease	10	84.29±0.33	87.64±0.15	85.97±0.15	54.76±0.25	89.19±0.10
RandomForest	miRNA-Gene	1	65.74±0.19	66.61±0.13	66.18±0.05	66.03±0.09	66.17±0.05
RandomForest	miRNA-Gene	10	65.74±0.19	70.56±0.14	68.15±0.03	28.57±0.03	76.33±0.08
RandomForest	miRNA-Phenotype	1	88.65±0.61	75.30±0.54	81.97±0.13	83.10±0.17	81.89±0.13
RandomForest	miRNA-Phenotype	10	88.65±0.61	86.88±0.41	87.77±0.19	55.44±0.51	89.06±0.24

Table S25: Results of the DT and RF models for specific edge prediction with 10 dimensions embeddings obtained from Node2Vec Skip-Gram BFS on miRNAdisease$_v$.

Model	Type pair	UR	Pos BA(%)	Neg BA(%)	Overall BA(%)	F1(%)	F1-Weighted(%)
DecisionTree	miRNA-Disease	1	74.34±0.24	73.18±0.34	73.76±0.15	73.91±0.14	73.76±0.15
DecisionTree	miRNA-Disease	10	74.34±0.24	80.86±0.26	77.60±0.07	40.65±0.21	83.85±0.15
DecisionTree	miRNA-Gene	1	58.50±0.11	54.99±0.16	56.74±0.11	57.49±0.10	56.73±0.11
DecisionTree	miRNA-Gene	10	58.50±0.11	59.31±0.18	58.91±0.11	20.69±0.08	67.86±0.14
DecisionTree	miRNA-Phenotype	1	76.96±1.05	74.91±0.23	75.94±0.52	76.18±0.64	75.93±0.52
DecisionTree	miRNA-Phenotype	10	76.96±1.05	85.13±0.15	81.04±0.51	47.26±0.49	86.87±0.12
RandomForest	miRNA-Disease	1	83.83±0.78	78.41±0.36	81.12±0.26	81.62±0.34	81.11±0.25
RandomForest	miRNA-Disease	10	83.83±0.78	87.41±0.19	85.62±0.32	54.14±0.22	89.00±0.09
RandomForest	miRNA-Gene	1	66.07±0.13	61.24±0.17	63.65±0.07	64.51±0.07	63.63±0.07
RandomForest	miRNA-Gene	10	66.07±0.13	65.67±0.17	65.87±0.05	25.94±0.05	72.98±0.11
RandomForest	miRNA-Phenotype	1	88.05±0.42	75.66±0.33	81.85±0.24	82.91±0.24	81.78±0.24
RandomForest	miRNA-Phenotype	10	88.05±0.42	86.80±0.48	87.42±0.22	55.04±0.74	88.95±0.31

S6.2 MiRNAdiseaseSO View

The Balanced Accuracy (BA) results for the specific edge prediction task on the type pairs are reported in table S26 for LINE and table S27 for Node2Vec.

Table S26: Results of the DT and RF models for specific edge prediction with 10 dimensions embeddings obtained from LINE on miRNAdiseaseSO$_v$.

Model	Type pair	UR	Pos BA(%)	Neg BA(%)	Overall BA(%)	F1(%)	F1-Weighted(%)
DecisionTree	miRNA-Disease	1	76.07±0.26	73.63±0.28	74.85±0.24	75.15±0.23	74.84±0.24
DecisionTree	miRNA-Disease	10	76.07±0.26	81.39±0.27	78.73±0.23	42.01±0.38	84.34±0.19
DecisionTree	miRNA-Gene	1	59.53±0.10	57.64±0.13	58.58±0.05	58.97±0.05	58.58±0.05
DecisionTree	miRNA-Gene	10	59.53±0.10	61.74±0.06	60.63±0.04	21.96±0.03	69.70±0.04
DecisionTree	miRNA-Phenotype	1	76.85±0.68	75.54±0.53	76.20±0.45	76.35±0.48	76.20±0.45
DecisionTree	miRNA-Phenotype	10	76.85±0.68	85.91±0.24	81.38±0.37	48.38±0.50	87.38±0.17
RandomForest	miRNA-Disease	1	84.14±0.40	78.64±0.18	81.39±0.15	81.89±0.19	81.37±0.15
RandomForest	miRNA-Disease	10	84.14±0.40	87.70±0.20	85.92±0.15	54.79±0.26	89.22±0.11
RandomForest	miRNA-Gene	1	65.96±0.18	66.37±0.11	66.16±0.05	66.09±0.09	66.16±0.05
RandomForest	miRNA-Gene	10	65.96±0.18	70.37±0.08	68.17±0.06	28.54±0.04	76.22±0.05
RandomForest	miRNA-Phenotype	1	87.35±0.22	76.52±0.56	81.94±0.24	82.86±0.19	81.88±0.25
RandomForest	miRNA-Phenotype	10	87.35±0.22	87.83±0.10	87.59±0.09	56.52±0.15	89.59±0.06

Table S27: Results of the DT and RF models for specific edge prediction with 10 dimensions embeddings obtained from Node2Vec Skip-Gram BFS on miRNAdiseaseSO$_v$.

Model	Type pair	UR	Pos BA(%)	Neg BA(%)	Overall BA(%)	F1(%)	F1-Weighted(%)
DecisionTree	miRNA-Disease	1	75.28±0.44	73.14±0.33	74.21±0.26	74.48±0.29	74.21±0.26
DecisionTree	miRNA-Disease	10	75.28±0.44	80.95±0.30	78.11±0.27	41.16±0.39	83.98±0.20
DecisionTree	miRNA-Gene	1	58.70±0.32	54.95±0.17	56.82±0.11	57.62±0.19	56.81±0.11
DecisionTree	miRNA-Gene	10	58.70±0.32	59.34±0.18	59.02±0.12	20.77±0.07	67.89±0.12
DecisionTree	miRNA-Phenotype	1	75.75±0.53	75.12±0.78	75.43±0.38	75.51±0.35	75.43±0.38
DecisionTree	miRNA-Phenotype	10	75.75±0.53	85.68±0.25	80.71±0.28	47.50±0.42	87.14±0.17
RandomForest	miRNA-Disease	1	84.01±0.62	78.47±0.17	81.24±0.34	81.74±0.38	81.22±0.34
RandomForest	miRNA-Disease	10	84.01±0.62	87.64±0.21	85.82±0.30	54.62±0.40	89.17±0.14
RandomForest	miRNA-Gene	1	65.91±0.14	61.64±0.20	63.78±0.06	64.53±0.05	63.76±0.06
RandomForest	miRNA-Gene	10	65.91±0.14	66.17±0.18	66.04±0.06	26.15±0.06	73.32±0.12
RandomForest	miRNA-Phenotype	1	87.28±0.36	76.45±0.82	81.87±0.29	82.80±0.21	81.81±0.30
RandomForest	miRNA-Phenotype	10	87.28±0.36	87.70±0.26	87.49±0.16	56.26±0.42	89.49±0.17

S6.3 GOdisease View

The Balanced Accuracy (BA) results for the specific edge prediction task on the type pairs are reported in table S28 for LINE and table S29 for Node2Vec.

Table S28: Results of the DT and RF models for specific edge prediction with 10 dimensions embeddings obtained from LINE on $GOdisease_v$.

Model	Type pair	UR	Pos BA(%)	Neg BA(%)	Overall BA(%)	F1(%)	F1-Weighted(%)
DecisionTree	Protein-GO	1	44.03±4.53	60.83±4.20	52.43±3.13	48.02±3.96	52.06±3.19
DecisionTree	Protein-GO	10	44.03±4.53	65.10±2.78	54.57±2.51	17.89±1.83	70.95±1.89
DecisionTree	lncRNA-Disease	1	65.65±0.57	63.16±0.73	64.41±0.57	64.85±0.54	64.40±0.57
DecisionTree	lncRNA-Disease	10	65.65±0.57	70.06±0.45	67.86±0.44	28.24±0.41	75.99±0.33
DecisionTree	lncRNA-GO	1	73.24±0.60	77.28±0.21	75.26±0.32	74.75±0.40	75.25±0.33
DecisionTree	lncRNA-GO	10	73.24±0.60	83.22±0.50	78.23±0.45	42.96±0.77	85.30±0.35
DecisionTree	lncRNA-Phenotype	1	59.55±1.95	57.13±1.33	58.34±0.98	58.83±1.28	58.33±0.97
DecisionTree	lncRNA-Phenotype	10	59.55±1.95	62.56±1.09	61.05±0.53	22.30±0.30	70.30±0.66
DecisionTree	miRNA-GO	1	71.11±0.33	69.97±0.29	70.54±0.22	70.71±0.24	70.54±0.22
DecisionTree	miRNA-GO	10	71.11±0.33	75.03±0.17	73.07±0.17	33.79±0.16	79.74±0.11
DecisionTree	miRNA-Gene	1	67.33±0.08	65.15±0.10	66.24±0.06	66.60±0.06	66.23±0.06
DecisionTree	miRNA-Gene	10	67.33±0.08	69.47±0.06	68.40±0.06	28.49±0.06	75.71±0.05
DecisionTree	miRNA-Phenotype	1	76.85±0.25	76.55±0.30	76.70±0.16	76.74±0.16	76.70±0.16
DecisionTree	miRNA-Phenotype	10	76.85±0.25	86.70±0.11	81.78±0.15	49.61±0.24	87.91±0.08
RandomForest	Protein-GO	1	36.29±4.05	75.09±1.29	55.69±2.52	44.96±4.22	53.93±2.92
RandomForest	Protein-GO	10	36.29±4.05	79.44±2.28	57.86±2.13	21.26±2.37	79.65±1.38
RandomForest	lncRNA-Disease	1	72.92±0.09	71.16±0.85	72.04±0.45	72.29±0.34	72.04±0.46
RandomForest	lncRNA-Disease	10	72.92±0.09	80.54±0.45	76.73±0.25	39.68±0.50	83.52±0.30
RandomForest	lncRNA-GO	1	79.46±1.15	81.49±0.47	80.47±0.70	80.27±0.79	80.47±0.70
RandomForest	lncRNA-GO	10	79.46±1.15	88.79±0.15	84.12±0.62	54.50±0.76	89.54±0.18
RandomForest	lncRNA-Phenotype	1	64.02±0.92	63.44±2.12	63.73±1.01	63.83±0.75	63.72±1.01
RandomForest	lncRNA-Phenotype	10	64.02±0.92	70.55±0.84	67.29±0.27	27.93±0.30	76.20±0.51
RandomForest	miRNA-GO	1	75.46±0.52	79.86±0.22	77.66±0.33	77.16±0.38	77.65±0.33
RandomForest	miRNA-GO	10	75.46±0.52	85.62±0.08	80.54±0.29	47.27±0.35	87.07±0.09
RandomForest	miRNA-Gene	1	72.30±0.16	74.41±0.13	73.36±0.11	73.07±0.12	73.35±0.11
RandomForest	miRNA-Gene	10	72.30±0.16	79.80±0.14	76.05±0.09	38.63±0.15	82.98±0.09
RandomForest	miRNA-Phenotype	1	88.09±0.21	76.49±0.29	82.29±0.19	83.26±0.17	82.23±0.19
RandomForest	miRNA-Phenotype	10	88.09±0.21	87.62±0.14	87.85±0.12	56.48±0.27	89.51±0.10

Table S29: Results of the DT and RF models for specific edge prediction with 10 dimensions embeddings obtained from Node2Vec Skip-Gram BFS on $GOdisease_v$.

Model	Type pair	UR	Pos BA(%)	Neg BA(%)	Overall BA(%)	F1(%)	F1-Weighted(%)
DecisionTree	Protein-GO	1	45.93±4.16	63.82±5.21	54.88±3.45	50.42±3.78	54.48±3.43
DecisionTree	Protein-GO	10	45.93±4.16	68.13±1.70	57.03±2.05	19.77±1.60	73.17±1.11
DecisionTree	lncRNA-Disease	1	65.45±0.65	63.78±0.50	64.61±0.36	64.91±0.43	64.61±0.36
DecisionTree	lncRNA-Disease	10	65.45±0.65	70.65±0.31	68.05±0.35	28.52±0.29	76.37±0.21
DecisionTree	lncRNA-GO	1	56.75±3.79	87.27±1.18	72.01±1.64	66.92±2.75	71.32±1.87
DecisionTree	lncRNA-GO	10	56.75±3.79	89.24±1.05	73.00±1.86	42.95±2.56	87.73±0.71
DecisionTree	lncRNA-Phenotype	1	58.57±1.30	56.27±1.10	57.42±0.81	57.90±0.94	57.41±0.81
DecisionTree	lncRNA-Phenotype	10	58.57±1.30	62.10±0.35	60.33±0.63	21.79±0.42	69.90±0.25
DecisionTree	miRNA-GO	1	69.54±0.43	66.09±0.99	67.81±0.58	68.36±0.48	67.80±0.58
DecisionTree	miRNA-GO	10	69.54±0.43	71.71±0.85	70.62±0.53	30.75±0.65	77.40±0.58
DecisionTree	miRNA-Gene	1	64.31±0.76	59.68±1.38	62.00±1.05	62.86±0.92	61.98±1.06
DecisionTree	miRNA-Gene	10	64.31±0.76	64.69±1.26	64.50±1.00	24.87±0.87	72.16±0.94
DecisionTree	miRNA-Phenotype	1	75.29±0.68	75.70±0.30	75.50±0.37	75.44±0.44	75.50±0.37
DecisionTree	miRNA-Phenotype	10	75.29±0.68	86.14±0.26	80.72±0.23	47.97±0.17	87.40±0.12
RandomForest	Protein-GO	1	38.78±5.62	79.47±4.26	59.12±4.70	48.62±6.34	57.33±5.01
RandomForest	Protein-GO	10	38.78±5.62	80.71±3.48	59.74±4.46	23.75±5.42	80.68±2.63
RandomForest	lncRNA-Disease	1	73.22±0.43	72.23±0.54	72.72±0.21	72.86±0.21	72.72±0.21
RandomForest	lncRNA-Disease	10	73.22±0.43	81.62±0.29	77.42±0.13	41.02±0.22	84.25±0.16
RandomForest	lncRNA-GO	1	60.70±1.77	89.89±0.53	75.29±1.02	71.06±1.44	74.75±1.09
RandomForest	lncRNA-GO	10	60.70±1.77	93.66±0.65	77.18±1.09	54.21±2.38	91.11±0.57
RandomForest	lncRNA-Phenotype	1	63.27±1.11	64.75±1.54	64.01±0.75	63.74±0.73	64.01±0.75
RandomForest	lncRNA-Phenotype	10	63.27±1.11	71.80±0.62	67.54±0.56	28.43±0.49	76.98±0.40
RandomForest	miRNA-GO	1	73.86±0.29	75.26±2.08	74.56±0.99	74.39±0.73	74.56±0.98
RandomForest	miRNA-GO	10	73.86±0.29	82.16±1.58	78.01±0.75	42.00±1.95	84.66±1.02
RandomForest	miRNA-Gene	1	69.37±1.15	67.82±2.21	68.60±1.61	68.85±1.45	68.59±1.61
RandomForest	miRNA-Gene	10	69.37±1.15	74.97±1.82	72.17±1.40	33.12±1.90	79.57±1.27
RandomForest	miRNA-Phenotype	1	87.31±0.55	76.45±0.43	81.88±0.26	82.81±0.28	81.82±0.26
RandomForest	miRNA-Phenotype	10	87.31±0.55	87.90±0.30	87.60±0.18	56.64±0.37	89.63±0.17

S6.4 PiRNAdisease View

The Balanced Accuracy (BA) results for the specific edge prediction task on the type pairs are reported in table S30 for LINE and table S31 for Node2Vec.

Table S30: Results of the DT and RF models for specific edge prediction with 10 dimensions embeddings obtained from LINE on piRNAdisease$_v$.

Model	Type pair	UR	Pos BA(%)	Neg BA(%)	Overall BA(%)	F1(%)	F1-Weighted(%)
DecisionTree	piRNA-Disease	1	90.50±2.45	95.59±0.33	93.04±1.20	92.85±1.32	93.04±1.20
DecisionTree	piRNA-Disease	10	90.50±2.45	95.68±0.19	93.09±1.26	77.44±1.58	95.51±0.30
DecisionTree	piRNA-Phenotype	1	N/A[1]	N/A[1]	N/A[1]	N/A[1]	N/A[1]
DecisionTree	piRNA-Phenotype	10	N/A[1]	N/A[1]	N/A[1]	N/A[1]	N/A[1]
RandomForest	piRNA-Disease	1	89.88±3.12	96.33±0.49	93.11±1.50	92.86±1.66	93.10±1.51
RandomForest	piRNA-Disease	10	89.88±3.12	96.76±0.50	93.32±1.51	80.90±2.17	96.31±0.44
RandomForest	piRNA-Phenotype	1	N/A[1]	N/A[1]	N/A[1]	N/A[1]	N/A[1]
RandomForest	piRNA-Phenotype	10	N/A[1]	N/A[1]	N/A[1]	N/A[1]	N/A[1]

[1] The data is not available because the model failed to generate a negative training graph with enough edges after the set number of iterations.

Table S31: Results of the DT and RF models for specific edge prediction with 10 dimensions embeddings obtained from Node2Vec Skip-Gram BFS on piRNAdisease$_v$.

Model	Type pair	UR	Pos BA(%)	Neg BA(%)	Overall BA(%)	F1(%)	F1-Weighted(%)
DecisionTree	piRNA-Disease	1	83.38±4.11	99.03±0.29	91.20±1.98	90.41±2.36	91.14±2.02
DecisionTree	piRNA-Disease	10	83.38±4.11	99.04±0.35	91.21±2.02	86.39±2.51	97.58±0.44
DecisionTree	piRNA-Phenotype	1	N/A[1]	N/A[1]	N/A[1]	N/A[1]	N/A[1]
DecisionTree	piRNA-Phenotype	10	N/A[1]	N/A[1]	N/A[1]	N/A[1]	N/A[1]
RandomForest	piRNA-Disease	1	83.36±4.12	99.70±0.09	91.53±2.04	90.73±2.42	91.46±2.08
RandomForest	piRNA-Disease	10	83.36±4.12	99.71±0.09	91.53±2.02	89.47±2.09	98.16±0.34
RandomForest	piRNA-Phenotype	1	N/A[1]	N/A[1]	N/A[1]	N/A[1]	N/A[1]
RandomForest	piRNA-Phenotype	10	N/A[1]	N/A[1]	N/A[1]	N/A[1]	N/A[1]

[1] The data is not available because the model failed to generate a negative training graph with enough edges after the set number of iterations.

S6.5 PiRNAdiseaseGO View

The Balanced Accuracy (BA) results for the specific edge prediction task on the type pairs are reported in table S32 for LINE and table S33 for Node2Vec.

S6.6 ChemicalDisease View

The Balanced Accuracy (BA) results for the specific edge prediction task on the type pairs are reported in table S34 for LINE and table S35 for Node2Vec.

Table S32: Results of the DT and RF models for specific edge prediction with 10 dimensions embeddings obtained from LINE on piRNAdiseaseGO$_v$.

Model	Type pair	UR	Pos BA(%)	Neg BA(%)	Overall BA(%)	F1(%)	F1-Weighted(%)
DecisionTree	piRNA-Disease	1	91.46±2.21	93.95±0.56	92.71±0.91	92.60±1.00	92.70±0.91
DecisionTree	piRNA-Disease	10	91.46±2.21	95.01±0.43	93.24±1.01	75.81±1.26	95.09±0.29
DecisionTree	piRNA-Phenotype	1	N/A[1]	N/A[1]	N/A[1]	N/A[1]	N/A[1]
DecisionTree	piRNA-Phenotype	10	N/A[1]	N/A[1]	N/A[1]	N/A[1]	N/A[1]
RandomForest	piRNA-Disease	1	92.05±2.45	94.34±0.60	93.20±0.93	93.11±1.03	93.20±0.93
RandomForest	piRNA-Disease	10	92.05±2.45	95.61±0.38	93.83±1.05	78.01±0.43	95.60±0.11
RandomForest	piRNA-Phenotype	1	N/A[1]	N/A[1]	N/A[1]	N/A[1]	N/A[1]
RandomForest	piRNA-Phenotype	10	N/A[1]	N/A[1]	N/A[1]	N/A[1]	N/A[1]

[1] The data is not available because the model failed to generate a negative training graph with enough edges after the set number of iterations.

Table S33: Results of the DT and RF models for specific edge prediction with 10 dimensions embeddings obtained from Node2Vec Skip-Gram BFS on piRNAdiseaseGO$_v$.

Model	Type pair	UR	Pos BA(%)	Neg BA(%)	Overall BA(%)	F1(%)	F1-Weighted(%)
DecisionTree	piRNA-Disease	1	83.17±3.02	98.71±0.19	90.94±1.51	90.15±1.77	90.88±1.54
DecisionTree	piRNA-Disease	10	83.17±3.02	98.92±0.11	91.04±1.52	85.73±1.93	97.45±0.33
DecisionTree	piRNA-Phenotype	1	N/A[1]	N/A[1]	N/A[1]	N/A[1]	N/A[1]
DecisionTree	piRNA-Phenotype	10	N/A[1]	N/A[1]	N/A[1]	N/A[1]	N/A[1]
RandomForest	piRNA-Disease	1	82.28±2.56	99.55±0.10	90.92±1.30	90.04±1.54	90.85±1.32
RandomForest	piRNA-Disease	10	82.28±2.56	99.64±0.04	90.96±1.28	88.51±1.57	97.99±0.26
RandomForest	piRNA-Phenotype	1	N/A[1]	N/A[1]	N/A[1]	N/A[1]	N/A[1]
RandomForest	piRNA-Phenotype	10	N/A[1]	N/A[1]	N/A[1]	N/A[1]	N/A[1]

[1] The data is not available because the model failed to generate a negative training graph with enough edges after the set number of iterations.

Table S34: Results of the DT and RF models for specific edge prediction with 10 dimensions embeddings obtained from LINE on $\mathrm{chemicalDisease_v}$.

Model	Type pair	UR	Pos BA(%)	Neg BA(%)	Overall BA(%)	F1(%)	F1-Weighted(%)
DecisionTree	Chemical-Disease	1	75.44±0.11	74.57±0.16	75.00±0.09	75.11±0.09	75.00±0.09
DecisionTree	Chemical-Disease	10	75.44±0.11	77.75±0.12	76.60±0.09	37.92±0.14	81.89±0.08
DecisionTree	Gene-Disease	1	61.02±0.88	66.53±0.91	63.78±0.86	62.75±0.88	63.75±0.86
DecisionTree	Gene-Disease	10	61.02±0.88	69.66±0.54	65.34±0.68	26.28±0.60	75.36±0.42
DecisionTree	lncRNA-Chemical	1	62.55±3.29	60.15±2.40	61.35±1.87	61.78±2.24	61.33±1.86
DecisionTree	lncRNA-Chemical	10	62.55±3.29	70.17±1.65	66.36±0.96	27.14±0.54	75.82±0.90
DecisionTree	lncRNA-Disease	1	72.72±0.72	71.88±0.70	72.30±0.33	72.42±0.38	72.30±0.33
DecisionTree	lncRNA-Disease	10	72.72±0.72	76.19±0.49	74.45±0.31	35.40±0.37	80.64±0.30
DecisionTree	miRNA-Chemical	1	59.86±1.37	54.60±1.40	57.23±0.90	58.32±0.97	57.20±0.90
DecisionTree	miRNA-Chemical	10	59.86±1.37	61.54±1.30	60.70±0.78	22.00±0.58	69.58±0.92
DecisionTree	miRNA-Disease	1	75.45±0.30	72.77±0.17	74.11±0.14	74.45±0.18	74.10±0.14
DecisionTree	miRNA-Disease	10	75.45±0.30	81.06±0.29	78.26±0.24	41.37±0.40	84.07±0.20
RandomForest	Chemical-Disease	1	81.76±0.21	83.31±0.38	82.53±0.17	82.40±0.15	82.53±0.17
RandomForest	Chemical-Disease	10	81.76±0.21	86.67±0.37	84.22±0.15	51.91±0.56	88.32±0.24
RandomForest	Gene-Disease	1	65.43±1.04	77.64±0.54	71.54±0.74	69.69±0.88	71.43±0.75
RandomForest	Gene-Disease	10	65.43±1.04	79.58±0.43	72.50±0.52	35.40±0.56	82.27±0.28
RandomForest	lncRNA-Chemical	1	69.76±2.87	63.11±0.95	66.43±1.79	67.50±2.07	66.39±1.77
RandomForest	lncRNA-Chemical	10	69.76±2.87	78.17±1.13	73.96±1.20	35.95±1.07	81.70±0.64
RandomForest	lncRNA-Disease	1	81.39±0.74	76.77±0.35	79.08±0.26	79.55±0.34	79.07±0.26
RandomForest	lncRNA-Disease	10	81.39±0.74	81.52±0.12	81.45±0.33	44.45±0.24	84.86±0.06
RandomForest	miRNA-Chemical	1	62.46±1.80	61.30±2.48	61.88±0.63	62.09±0.68	61.87±0.64
RandomForest	miRNA-Chemical	10	62.46±1.80	70.24±2.13	66.35±0.30	27.18±0.63	75.86±1.31
RandomForest	miRNA-Disease	1	83.38±0.32	77.26±0.28	80.32±0.14	80.90±0.15	80.30±0.14
RandomForest	miRNA-Disease	10	83.38±0.32	86.83±0.18	85.10±0.14	52.93±0.28	88.57±0.11

Table S35: Results of the DT and RF models for specific edge prediction with 10 dimensions embeddings obtained from Node2Vec Skip-Gram BFS on chemicalDisease$_v$.

Model	Type pair	UR	Pos BA(%)	Neg BA(%)	Overall BA(%)	F1(%)	F1-Weighted(%)
DecisionTree	Chemical-Disease	1	74.03±0.10	73.85±0.32	73.94±0.18	73.96±0.15	73.94±0.18
DecisionTree	Chemical-Disease	10	74.03±0.10	76.92±0.25	75.47±0.15	36.57±0.25	81.23±0.17
DecisionTree	Gene-Disease	1	58.76±0.72	62.14±0.70	60.45±0.27	59.77±0.39	60.44±0.27
DecisionTree	Gene-Disease	10	58.76±0.72	65.40±0.56	62.08±0.29	23.28±0.22	72.26±0.36
DecisionTree	lncRNA-Chemical	1	49.28±4.78	62.35±3.05	55.81±3.89	52.69±4.59	55.62±3.96
DecisionTree	lncRNA-Chemical	10	49.28±4.78	71.94±1.57	60.61±2.50	22.94±2.16	75.97±1.09
DecisionTree	lncRNA-Disease	1	69.07±0.89	73.13±0.52	71.10±0.39	70.50±0.52	71.08±0.39
DecisionTree	lncRNA-Disease	10	69.07±0.89	77.39±0.29	73.23±0.40	34.96±0.35	81.14±0.17
DecisionTree	miRNA-Chemical	1	58.96±2.28	53.71±1.09	56.33±0.73	57.43±1.33	56.29±0.71
DecisionTree	miRNA-Chemical	10	58.96±2.28	60.72±1.42	59.84±0.62	21.37±0.37	68.92±0.91
DecisionTree	miRNA-Disease	1	74.90±0.45	72.46±0.41	73.68±0.17	74.00±0.21	73.68±0.17
DecisionTree	miRNA-Disease	10	74.90±0.45	80.53±0.28	77.72±0.23	40.53±0.32	83.68±0.18
RandomForest	Chemical-Disease	1	80.69±0.15	85.64±0.26	83.16±0.19	82.74±0.18	83.15±0.19
RandomForest	Chemical-Disease	10	80.69±0.15	88.84±0.17	84.76±0.14	55.22±0.36	89.69±0.12
RandomForest	Gene-Disease	1	61.39±0.81	77.56±0.34	69.48±0.52	66.79±0.66	69.27±0.54
RandomForest	Gene-Disease	10	61.39±0.81	79.62±0.29	70.50±0.38	33.62±0.37	81.95±0.18
RandomForest	lncRNA-Chemical	1	52.50±5.75	68.18±1.44	60.34±3.10	56.86±4.43	60.06±3.23
RandomForest	lncRNA-Chemical	10	52.50±5.75	80.84±1.20	66.67±2.34	30.45±1.85	81.96±0.38
RandomForest	lncRNA-Disease	1	78.24±0.49	79.37±0.42	78.81±0.18	78.68±0.22	78.80±0.18
RandomForest	lncRNA-Disease	10	78.24±0.49	84.28±0.35	81.26±0.26	46.65±0.48	86.43±0.22
RandomForest	miRNA-Chemical	1	62.92±1.40	60.97±1.14	61.95±0.96	62.31±1.06	61.94±0.96
RandomForest	miRNA-Chemical	10	62.92±1.40	70.66±0.90	66.79±0.39	27.58±0.29	76.19±0.52
RandomForest	miRNA-Disease	1	83.75±0.43	78.11±0.58	80.93±0.19	81.45±0.17	80.91±0.20
RandomForest	miRNA-Disease	10	83.75±0.43	87.58±0.27	85.66±0.13	54.40±0.33	89.11±0.15

S6.7 CellAnatomy View

The Balanced Accuracy (BA) results for the specific edge prediction task on the type pairs are reported in table S36 for LINE and table S37 for Node2Vec.

Table S36: Results of the DT and RF models for specific edge prediction with 10 dimensions embeddings obtained from LINE on cellAnatomy$_{\mathrm{v}}$.

Model	Type pair	UR	Pos BA(%)	Neg BA(%)	Overall BA(%)	F1(%)	F1-Weighted(%)
DecisionTree	Protein-Anatomy	1	64.94±0.57	67.88±0.54	66.41±0.24	65.91±0.30	66.40±0.24
DecisionTree	Protein-Anatomy	10	64.94±0.57	71.05±0.43	68.00±0.21	28.58±0.20	76.61±0.26
DecisionTree	Protein-Cell	1	67.15±0.71	64.87±0.78	66.01±0.66	66.39±0.65	66.00±0.66
DecisionTree	Protein-Cell	10	67.15±0.71	75.54±0.81	71.34±0.72	32.63±0.87	79.77±0.58
DecisionTree	Protein-Protein	1	77.46±0.32	84.21±0.19	80.83±0.20	80.17±0.23	80.81±0.20
DecisionTree	Protein-Protein	10	77.46±0.32	85.62±0.13	81.54±0.16	48.22±0.21	87.24±0.08
DecisionTree	lncRNA-Anatomy	1	81.05±0.55	79.86±0.38	80.46±0.33	80.57±0.35	80.46±0.33
DecisionTree	lncRNA-Anatomy	10	N/A[1]	N/A[1]	N/A[1]	N/A[1]	N/A[1]
DecisionTree	lncRNA-Protein	1	68.92±0.30	66.07±0.28	67.50±0.24	67.95±0.24	67.49±0.24
DecisionTree	lncRNA-Protein	10	68.92±0.30	79.17±0.32	74.05±0.25	36.55±0.37	82.29±0.22
DecisionTree	miRNA-Protein	1	70.57±0.53	67.57±0.26	69.07±0.21	69.52±0.29	69.06±0.21
DecisionTree	miRNA-Protein	10	70.57±0.53	80.80±0.13	75.68±0.22	38.93±0.16	83.49±0.06
RandomForest	Protein-Anatomy	1	64.84±0.41	78.82±0.77	71.83±0.35	69.71±0.31	71.69±0.34
RandomForest	Protein-Anatomy	10	64.84±0.41	81.36±0.28	73.10±0.10	36.92±0.16	83.37±0.15
RandomForest	Protein-Cell	1	69.10±0.89	73.24±0.81	71.17±0.68	70.56±0.73	71.16±0.68
RandomForest	Protein-Cell	10	69.10±0.89	85.57±0.49	77.34±0.52	44.11±0.86	86.48±0.34
RandomForest	Protein-Protein	1	79.86±0.33	91.17±0.09	85.52±0.16	84.65±0.20	85.47±0.17
RandomForest	Protein-Protein	10	79.86±0.33	92.04±0.11	85.95±0.13	61.57±0.17	91.83±0.06
RandomForest	lncRNA-Anatomy	1	85.52±0.40	87.03±0.37	86.27±0.35	86.17±0.35	86.27±0.35
RandomForest	lncRNA-Anatomy	10	N/A[1]	N/A[1]	N/A[1]	N/A[1]	N/A[1]
RandomForest	lncRNA-Protein	1	78.49±0.35	70.53±0.53	74.51±0.43	75.48±0.39	74.47±0.43
RandomForest	lncRNA-Protein	10	78.49±0.35	86.57±0.38	82.53±0.35	50.19±0.75	87.96±0.28
RandomForest	miRNA-Protein	1	79.43±0.26	72.68±0.19	76.06±0.11	76.84±0.13	76.03±0.11
RandomForest	miRNA-Protein	10	79.43±0.26	87.91±0.19	83.67±0.07	52.90±0.23	88.95±0.11

[1] The data is not available because the model failed to generate a negative training graph with enough edges after the set number of iterations.

Table S37: Results of the DT and RF models for specific edge prediction with 10 dimensions embeddings obtained from Node2Vec Skip-Gram BFS on $cellAnatomy_v$.

Model	Type pair	UR	Pos BA(%)	Neg BA(%)	Overall BA(%)	F1(%)	F1-Weighted(%)
DecisionTree	Protein-Anatomy	1	66.98±0.74	74.28±1.89	70.63±1.12	69.52±0.96	70.59±1.11
DecisionTree	Protein-Anatomy	10	66.98±0.74	76.03±1.41	71.50±0.91	32.97±1.23	80.08±0.95
DecisionTree	Protein-Cell	1	67.52±0.96	67.62±3.17	67.57±1.79	67.57±1.40	67.57±1.79
DecisionTree	Protein-Cell	10	67.52±0.96	77.59±1.70	72.56±1.01	34.53±1.56	81.14±1.12
DecisionTree	Protein-Protein	1	71.41±1.27	82.21±1.23	76.81±1.23	75.49±1.31	76.74±1.24
DecisionTree	Protein-Protein	10	71.41±1.27	83.62±1.14	77.51±1.19	42.65±2.05	85.40±0.85
DecisionTree	lncRNA-Anatomy	1	80.34±0.39	79.96±0.68	80.15±0.43	80.19±0.40	80.15±0.43
DecisionTree	lncRNA-Anatomy	10	N/A[1]	N/A[1]	N/A[1]	N/A[1]	N/A[1]
DecisionTree	lncRNA-Protein	1	68.67±0.29	67.59±0.44	68.13±0.31	68.30±0.28	68.13±0.31
DecisionTree	lncRNA-Protein	10	68.67±0.29	80.18±0.32	74.42±0.22	37.43±0.35	82.93±0.21
DecisionTree	miRNA-Protein	1	70.47±0.31	67.92±0.38	69.20±0.24	69.59±0.24	69.19±0.24
DecisionTree	miRNA-Protein	10	70.47±0.31	80.40±0.37	75.44±0.28	38.47±0.46	83.22±0.25
RandomForest	Protein-Anatomy	1	68.33±0.82	85.15±1.03	76.74±0.88	74.60±0.93	76.57±0.88
RandomForest	Protein-Anatomy	10	68.33±0.82	86.17±0.85	77.25±0.73	44.60±1.48	86.80±0.60
RandomForest	Protein-Cell	1	71.25±3.40	74.85±0.88	73.05±1.80	72.53±2.28	73.03±1.82
RandomForest	Protein-Cell	10	71.25±3.40	86.12±0.48	78.69±1.61	45.95±1.53	87.03±0.33
RandomForest	Protein-Protein	1	76.12±0.83	92.31±0.66	84.22±0.70	82.83±0.78	84.11±0.71
RandomForest	Protein-Protein	10	76.12±0.83	92.97±0.61	84.55±0.67	61.81±1.91	92.14±0.50
RandomForest	lncRNA-Anatomy	1	85.19±0.30	87.24±0.99	86.21±0.49	86.07±0.44	86.21±0.49
RandomForest	lncRNA-Anatomy	10	N/A[1]	N/A[1]	N/A[1]	N/A[1]	N/A[1]
RandomForest	lncRNA-Protein	1	76.97±0.34	73.04±0.58	75.01±0.25	75.49±0.21	75.00±0.26
RandomForest	lncRNA-Protein	10	76.97±0.34	88.01±0.29	82.49±0.08	51.85±0.37	88.79±0.17
RandomForest	miRNA-Protein	1	78.81±0.52	73.93±0.52	76.37±0.30	76.93±0.31	76.35±0.30
RandomForest	miRNA-Protein	10	78.81±0.52	88.55±0.22	83.68±0.25	53.73±0.41	89.32±0.14

[1] The data is not available because the model failed to generate a negative training graph with enough edges after the set number of iterations.

S7 Implementation of the Unbiased Training Procedure

The edge prediction pipeline in GRAPE allows you to pass a positive training set, but it does not allow you to pass a negative training set; instead, the negative training set is generated internally.

According to the code published on the GRAPE GitHub repository, the positive training set used is the one passed as a parameter to the `fit` method. This method handles all the required training operations, so it also takes care of generating the negative training set.

The way the negative training set is generated can significantly impact the model's performance.

According to the repository, the negative training set is generated from the positive training set (`graph` in the code) using the following code:

```
negative_graph = graph.sample_negative_graph(
            number_of_negative_samples =
                number_of_negative_samples,
            only_from_same_component =
                True,
            random_state =
                self._random_state,
            use_scale_free_distribution =
                self._use_scale_free_distribution,
            sample_edge_types =
                len(edge_type_features) > 0,
        )
```

The graph variable contains the positive training graph and `sample_negative_graph` is a method used to generate the negative training set. This method can also accept two additional parameters: `source_node_types_names` and `destination_node_types_names` that are used to generate only some specific edges, but that are not specified in this case, so this method generates a negative training graph that has the same source and destination node types for the edges as the training set.

The main issue with this method is that the graph used to generate the negative training graph is the positive training graph given to the model, which represents only the positive training portion of the holdout set, rather than the full graph. This choice is problematic because some negative edges generated might actually be positive examples that are not included in the training set given to the model but are in the testing set that was left out. This could lead to false negatives that can confuse the predictor being trained, degrading its performance.

S7.1 Updating the Training Function

There are two ways to update the training function of a model to change how the training is performed:

- Starting from scratch: this requires reimplementing the model completely, while still ensuring compatibility with GRAPE graphs.
- Updating the classes/objects from GRAPE: this allows for a much faster development time and reduces the risk of errors.

We opted for the second way: updating the classes/objects of the edge prediction models from GRAPE to change the training behaviour.

The GRAPE classes method uses the `_fit` method internally to do the actual training, this method is defined as an abstract method in the abstract class `AbstractClassifierModel`, and it is overridden in the `SklearnLikeEdgePredictionAdapter` class where there is the code to do the actual training.

Figure S14 shows the GRAPE edge prediction models structure for the DT and RF models, along with some possible ways to update it to allow for a custom `_fit` function.

To update the edge prediction classes in GRAPE, we considered three options. The first option (on the right, yellow in figure S14) would be to update the class structure with a custom `SklearnLikeEdgePredictionAdapter` and override the `_fit` function with the custom one at that level to avoid having to create a custom class for each model. This option is difficult to implement because the class is part of a package and updating it does not directly update the subclasses, this would also change the behaviour of the whole edge prediction library branch, which might be unwanted behaviour as well.

The second option (down, blue in figure S14) would be to create a subclass of each edge prediction model we are interested in and override the `fit` function there. This option is easier to implement but requires creating a new class for each model we want to use.

The third option would be to update the instance of the class directly: it is possible to develop a function to update the instance of the edge prediction model, updating the `_fit` function to follow the required behaviour. This option is also easy to implement and does not require creating a subclass for each model, allowing it to be more general.

Both the second and third option were implemented, the outputs produced are the same.

The main changes to apply to the training function are:

1. Add an additional parameter or class attribute that contains the negative train graph generated externally.
2. Change the behavior of the training function to use the "externally" generated negative training graph.

For the experiments of this paper, the third option was chosen: updating the _fit function. The code is available on GitHub.

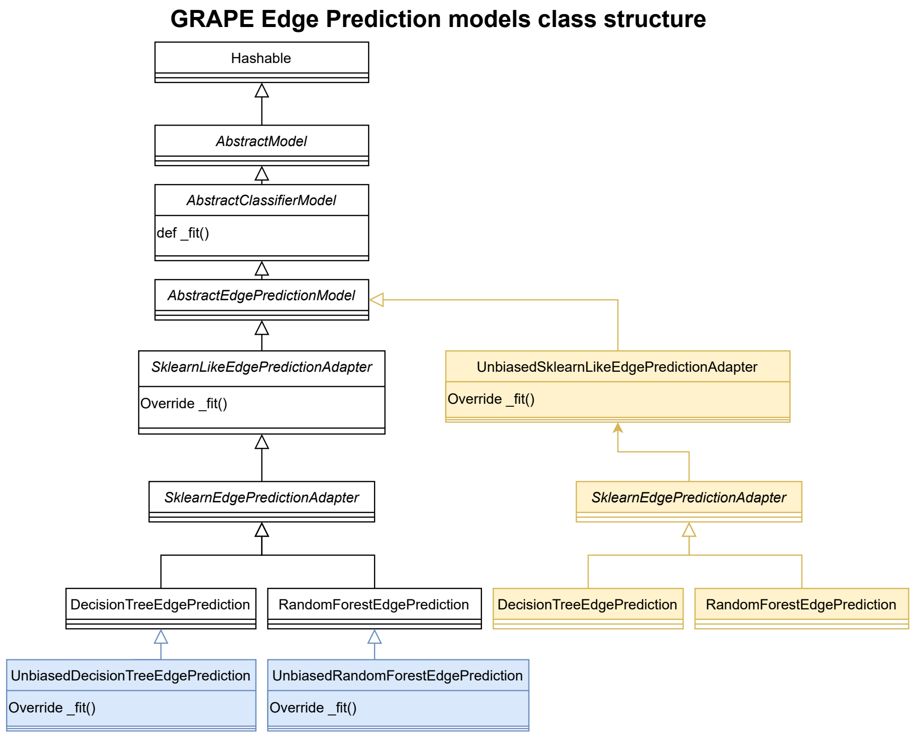

Figure S14: The class structure for the edge prediction models in GRAPE focused on the DT and RF models, with some possible extensions to allow for a custom training function. The classes on the right (yellow) show the option to override the `_fit` function at the general abstract class level, meanwhile the classes at the bottom (blue) show an alternative option, which is to create a custom class that extends each model and overrides the `fit` function as needed.

References

[1] Michael Ashburner, Catherine A. Ball, Judith A. Blake, et al. Gene ontology: tool for the unification of biology. *Nature Genetics*, 25(1):25–29, May 2000.

[2] Tiffany J. Callahan, Ignacio J. Tripodi, Adrianne L. Stefanski, et al. An open source knowledge graph ecosystem for the life sciences. *Scientific Data*, 11(1), April 2024.

[3] Karen Eilbeck, Suzanna E Lewis, Christopher J Mungall, et al. The sequence ontology: a tool for the unification of genome annotations. *Genome Biology*, 6(5), April 2005.

[4] Peng Xu, Qian Wu, Jian Yu, Yongsheng Rao, Zheng Kou, Gang Fang, Xiaolong Shi, Wenbin Liu, and Henry Han. A systematic way to infer the regulation relations of miRNAs on target genes and critical miRNAs in cancers. *Front. Genet.*, 11:278, March 2020.

[5] Qun Zhang, Yazhi Zhu, Xinyu Cao, Wenhui Tan, Jianglong Yu, Yaqiong Lu, Ran Kang, Xiaolan Wang, and Ermao Li. The epigenetic regulatory mechanism of PIWI/piRNAs in human cancers. *Molecular Cancer*, 22(1), March 2023.
